# The effect of mycophenolate mofetil on podocytes in nephrotoxic serum nephritis

**DOI:** 10.1038/s41598-023-41222-1

**Published:** 2023-08-29

**Authors:** A. Hackl, E. Nüsken, J. Voggel, S. E. D. Abo Zed, J. Binz-Lotter, D. Unnersjö-Jess, C. Müller, G. Fink, K. Bohl, E. Wiesner, P. Diefenhardt, C. Dafinger, H. Chen, M. Wohlfarth, R.-U. Müller, M. J. Hackl, B. Schermer, K.-D. Nüsken, L. T. Weber

**Affiliations:** 1 Department of Pediatrics, Faculty of Medicine, University Hospital Cologne, University of Cologne, Kerpener Street 62, 50937 Cologne, Germany; 2 CECAD, Faculty of Medicine, University Hospital Cologne, University of Cologne, Cologne, Germany; 3 Department 2 of Internal Medicine, Faculty of Medicine, University Hospital Cologne, University of Cologne, Cologne, Germany; 4 Department of Therapeutic Drug Monitoring, Pharmacology at the Laboratory Centre, Faculty of Medicine, University Hospital Cologne, University of Cologne, Cologne, Germany; 5 Center for Rare Kidney Diseases Cologne, Faculty of Medicine, University Hospital Cologne, University of Cologne, Cologne, Germany

**Keywords:** Diseases, Medical research, Nephrology

## Abstract

Mycophenolate mofetil (MMF) is applied in proteinuric kidney diseases, but the exact mechanism of its effect on podocytes is still unknown. Our previous in vitro experiments suggested that MMF can ameliorate podocyte damage via restoration of the Ca^2+^-actin cytoskeleton axis. The goal of this study was to characterize podocyte biology during MMF treatment in nephrotoxic serum (NTS) nephritis (NTN). NTN was induced in three-week old wild-type mice. On day 3, half of the mice were treated with MMF (100 mg/kgBW/d p.o.) for one week. On day 10, we performed proteomic analysis of glomeruli as well as super-resolution imaging of the slit diaphragm. For multiphoton imaging of Ca^2+^ concentration ([Ca^2+^]_i_), the experimental design was repeated in mice expressing podocyte-specific Ca^2+^ sensor. MMF ameliorated the proteinuria and crescent formation induced by NTS. We identified significant changes in the abundance of proteins involved in Ca^2+^ signaling and actin cytoskeleton regulation, which was further confirmed by direct [Ca^2+^]_i_ imaging in podocytes showing decreased Ca^2+^ levels after MMF treatment. This was associated with a tendency to restoration of podocyte foot process structure. Here, we provide evidence that MPA has a substantial direct effect on podocytes. MMF contributes to improvement of [Ca^2+^]_i_ and amelioration of the disorganized actin cytoskeleton in podocytes. These data extend the knowledge of direct effects of immunosuppressants on podocytes that may contribute to a more effective treatment of proteinuric glomerulopathies with the least possible side effects.

## Introduction

Glomerulopathies account for 5–14% of children with chronic kidney disease and 15–29% of children with kidney failure worldwide^1^. Many patients with glomerulopathies can only be maintained in remission using longstanding treatment with steroids. However, the use of this drug for prolonged time results in serious adverse effects. Therefore, especially pediatricians have been investigating alternative steroid-sparing therapeutic options such as mycophenolate mofetil (MMF) in this vulnerable population^2^.

MMF, the pro-drug of the biologically active mycophenolic acid (MPA), acts as a potent, reversible inhibitor of IMPDH, the key enzyme in de novo purine biosynthesis in proliferating lymphocytes, thereby suppressing cell-mediated immune responses and antibody formation^3^. The first study on kidney effects of MMF showed that it prevents the process of chronic allograft rejection by decreasing the expression of adhesion molecules and cytokines in glomeruli, thus preventing glomerulosclerosis^4^. In the past two decades, MMF has been established and widely used also in inflammatory glomerulopathies^5^.

Beyond the well characterized, immunosuppressive effect, we know little about how MMF directly acts on the kidney tissue, especially podocytes^6,7^. In diabetic nephropathy, lower apoptotic rate of podocytes and preserved expression of nephrin and podocin were observed in the MMF treated group^8^. This is in accordance with the results in a puromycin model where the inhibition of IMPDH restored the ATP pool, which retained nephrin and synaptopodin expression and stabilized the actin cytoskeleton^9^. Additionally, Fu et al. showed a direct effect of MMF in an immune-mediated kidney disease: MRL/lpr mice were treated with MMF. Subsequently, RNA sequencing performed on isolated kidney cortex showed a significant decrease of *Rac1* gene expression, which disrupts the physiological stress fiber formation, when activated^10^. In line with that our previous in vitro experiments performing RNA sequencing of a MPA treated podocyte cell line revealed an accumulation of the terms “actin cytoskeleton” and “regulation of small GTPase mediated signal transduction”*.* Additionally, also the terms “calcium channel activity” and “calcium ion transport” were enriched^11^. The rise in intracellular calcium concentration ([Ca^2+^]_i_) in podocytes^12^ can lead to acute reorganization of the actin cytoskeleton or even cytoskeletal collapse and proteinuria^13–16^. In the present study, we wanted to confirm, whether MMF is also able to ameliorate podocyte damage via restoration of the Ca^2+^- actin cytoskeleton axis in an in vivo model of nephrotoxic serum nephritis (NTN).

## Methods

### Animal model

**Figure 1:**
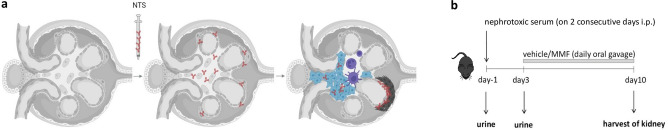
Panel (**a**) depicts the underlying pathomechanism of nephrotoxic serum nephritis. Nephrotoxic antibodies (heterologous sheep antibodies raised against rat whole glomeruli) (red) are deposited in the whole glomerulus including the subepithelial, subendothelial areas as well as mesangium leading histologically to mesangial proliferation (blue) and crescent formation (black) and clinically to immune-mediated glomerulopathy with proteinuria. At the time of harvest, glomerular cells are in a complex inflammatory environment (purple) with dominance of Th17 and Th1 cells accompanied by pro-inflammatory cDC2s and counterbalancing cDC1s cells. Created with BioRender.com Panel (**b**) shows the design and timeline of our study. Nephrotoxic serum nephritis was induced in three week old mice by intraperitoneal injection of nephrotoxic serum on two consecutive days. On day 3, after proteinuria induction, either vehicle or MMF in a dose of 100 mg/kgBW/d were administered orally for seven days and then mice were sacrificed. Mice without NTS induction served as control. NTS: nephrotoxic serum, MMF: mycophenolate mofetil.

C57BL6 mice as well as Pod:cre tg/wt GCaMP3 fl/fl mice in the C57BL6 genetic background were housed according to the standardized specific pathogen-free conditions in the University of Cologne animal facility. GCaMP3 mice were provided by JAX Laboratories (strain 014538), Pod:cre mice^17^ are from an established colony (> 10 years) in our vivarium. All animal experiments were performed in accordance with relevant guidelines and regulations provided by the LANUV NRW (Landesamt für Natur, Umwelt und Verbraucherschutz Nordrhein-Westfalen/State Agency for Nature, Environment and Consumer Protection North Rhine-Westphalia). The experimental protocol was examined and approved by the LANUV NRW (VSG81-02.04.2020.A052), which adhered to the 3R principles. Each mouse was considered as n = 1. Altogether 13 mice (5 females, 8 males) constituted the control group, 11 mice (7 females, 4 males) the NTS + veh group and 12 mice (7 females, 5 males) the NTS + MMF group. NTN was induced in three-week old mice (nephrogenesis is already completed, but they are not yet sexually mature) as follows: The animals received intraperitoneal injection of nephrotoxic serum (NTS; Probetex, San Antonio, USA) in a dose of 10 µl/g body weight (BW) on two consecutive days. From a pathogenic point of view, the historically coined term “nephrotoxic” is misleading since the serum induces an immune-mediated process. In detail, nephrotoxic antibodies (heterologous sheep antibodies raised against rat whole glomeruli) are deposited in the whole glomerulus including the subepithelial, subendothelial areas as well as mesangium. Subsequently, mesangial proliferation and crescent formation can be observed. Thus NTN serves as a model for immune-mediated glomerulopathy with proteinuria (Fig. 1a). In our study, all NTS-injected mice showed proteinuria on day 3. Mice without NTS induction served as control. Confounders were not controlled. On day 3, either water (NTS + veh) or MMF (Roche, Mannheim, Germany) in a dose of 100 mg/kgBW/d (NTS + MMF) were administered per oral gavage for seven days. They were then anesthetized with ketamine and xylazine followed by cardiac blood sampling and sacrificed by cardiac perfusion with cold phosphate-buffered saline (PBS). Depending on the further examination technique, mice were additionally perfused with 4% paraformaldehyde. The kidneys were removed and either put in melted agarose for acute kidney slices (AKS) or in 4% neutral buffered formalin for stimulated emission depletion (STED) imaging. Spot urine samples were taken before injection with NTS, on day 3 and the last day. The experimental design is depicted in Fig. 1b.

### Genotyping

Mice were genotyped using DNA isolated from ear biopsies. For Pod:cre tg/wt GCaMP3 fl/fl mice, DNA was amplified by PCR using REDTaq ReadyMix (Sigma-Aldrich, Taufkirchen, Germany) and visualized via gel electrophoresis. All primers used and the respective fragment size detected in gel electrophoresis are listed in Suppl. Table 1.

### Urine analysis

Quantification of the urinary albumin levels was performed with a mouse albumin ELISA kit (ICL/Dunn Labortechnik, Asbach, Germany) and creatinine levels were measured with a urinary creatinine kit (Biomol, Hamburg, Germany) according to the manufactor’s instructions.

### Histopathology

In kidneys, scheduled for histopathology, dehydration and embedding in paraffin was performed. Kidney tissue was cut and sections were transferred on glass slides. To assess glomerulosclerosis periodic acid–Schiff (PAS) staining was performed. Briefly, sections were deparaffinized in Xylene and subsequently rehydrated in a descending ethanol series. After incubation for 10 min each in 0.9% periodic acid (Carl Roth, Karslruhe, Germany), Schiff reagent (Merck, Darmstadt, Germany) and Mayer’s haematoxylin (Merck) the sections were dehydrated in an ascending ethanol series and xylene, and covered with HistoMount (National Diagnostics, Atlanta, USA). A NanoZoomer S360 (Hamamatsu, Geldern, Germany) was used to acquire images. To determine the extend of glomerular damage, the number of crescentic glomeruli was counted in a blinded manner. Crescents were defined as two or more cellular layers in the Bowman`s space. A minimum of 50 glomeruli were assessed per kidney^18^.

### Therapeutic drug monitoring

On the last day of the experiment, 24 h after the latest dose of MMF, NTS + MMF mice underwent a cheek punch procedure providing the blood sample for MPA-C_0_ of the therapeutic drug monitoring (TDM)_,_ followed directly by the last dose of MMF. After 30 min a terminal blood taking was conducted, which served as MPA-C_30_ of TDM. Accordingly, a limited sampling strategy was used. Estimated MPA–area under the concentration–time curve profiles (eMPA-AUC_0–24 h_) was derived from plasmatic MPA concentrations and calculated based on a bayesian-fitting approach with a two-compartment model using the pharmacokinetic software package MW/Pharm ++ (licensed version)^19^.

### Isolation of glomeruli and tandem mass spectrometry analysis

After sacrifice by cervical dislocation, wild-type mouse kidneys were perfused with Dynabeads to isolate glomeruli as described^20^. The isolated glomeruli were resuspended in 500 µL 1 × Hanks’ Balanced Salt Solution and centrifuged at 1500 rpm for 5 min at 4 °C, afterwards the supernatant was removed. Pellets were resuspended in 50 µL SP3 buffer with 5% SDS and again centrifuged at 15,000 g for 5 min at 4 °C. Afterwards, chromatin was degraded in a Bioruptor® sonicator. The reaction tubes were placed in a magnetic rack to remove the Dynabeads from the samples. The supernatant was boiled at 95 °C and then centrifuged at 15,000 g for 2 min at 4 °C. The protein concentration was determined by a standard procedure using a BCA-kit^21^. The concentration of each sample was adjusted to 4 µg using 1 × SP3 puffer. The glomerular proteome was analyzed by a mass spectrometry system containing a Q ExactivePlusOrbitrap and an EASY nLC 1000 with a C18 analytical column. The detailed procedure has been described elsewhere^22^. Statistical analysis was performed with the Perseus software (version 1.5.5.3)^23^ and the package limma in R^24^. A one-way ANOVA and a two-sample t-test were applied for the data set after filtering proteins for 3 valid values in at least one group. A detected protein was defined as valid, when at least two peptides of the protein were identified. Limma functions lmFit, eBayes and topTable were used with default settings on the filtered log_2_ transformed protein intensities to calculate *p* values and log_2_ fold changes. A *p* value of 0.05 was assumed to be significant without further correction for multiple testing. Within this, altered proteins with a log_2_ fold change (fc) of ≥|0.58| (fc ≥ 1.5) were categorized as relevantly altered. Proteins that reached these criteria were further analyzed. GraphPad Prism v.9.0.2, Uniprot protein knowledgebase and ShinyGO (v.0.75)^25^ were used for functional analysis.

### Ca^2+^ imaging with multiphoton microscopy on AKS

Murine kidneys of Pod:cre tg/wt GCaMP3 fl/fl mice were removed directly postmortem and then embedded in 4% low melting agarose at 37 °C. The kidney in agarose was cut in slices of 300 µm thickness and kept at 4 °C. For imaging, AKS was transferred to the microscope stage containing Krebs–Henseleit-Buffer at RT infused with 95% O_2_ and 5% CO_2_. The ex vivo images were acquired using an upright multiphoton microscope (TCSSP8 MP-OPO, Leica Microsystems, Wetzlar, Germany). The multiphoton laser (Chameleon VisionII, Coherent, Dieburg, Germany) was set to 940 nm, a 25 × water objective (numerical aperture of 0.95) was used and images were recorded applying a non-descanned hybrid detector with a FITC/TRITC (525/50) filter set. Z-stacks were taken in 2 µm steps. For analysis of Ca^2+^ levels in podocytes, ROIs were placed on maximum intensity projections of z-stacks of single glomeruli and the mean fluorescence intensity was quantified with ImageJ. At least twenty glomeruli per slice and two slices per animal were imaged and analyzed by an observer blinded to the treatment.

### Imaging of foot processes with STED microscopy and quantification of slit diaphragm (SD) length per area

The protocol of optical clearing and immunolabelling were performed as previously described^26,27^. Supp. Table 2 shows the antibodies used. The quantification of SD length followed previously described methods^27^ using an ImageJ macro, which can be found in GitHub gist: https://gist.github.com/github-martin/e699d18ae6cfa5b1a6aace15d3c3544c.

All methods are reported in accordance with ARRIVE guidelines.

## Results

**Figure 2:**
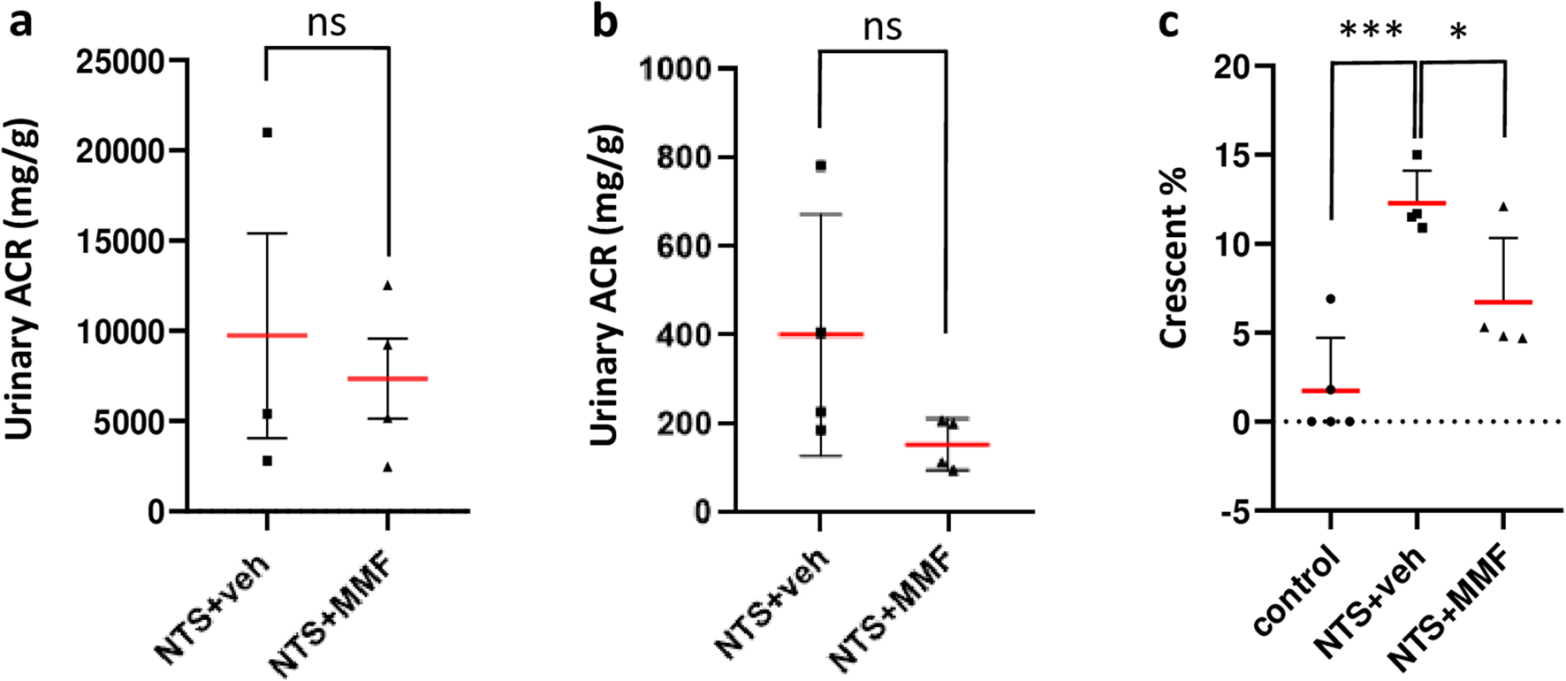
MMF therapy attenuates renal damage in NTN model. Panel (**a**) shows urinary albumin creatinine ratio on day 3. Proteinuria of NTS + veh and NTS + MMF did not differ significantly. Panel (**b**) shows urinary albumin creatinine ratio on day 10. One-week treatment with MMF in a dose of 100 mg/kgBW/d resulted in a moderate difference in reduction of proteinuria in nephrotoxic serum nephritis. Panel (**c**) depicts the percentage of crescentic glomeruli in each group. NTS + MMF animals presented with significant less glomerular crescent compared to NTS + veh mice. NTS + veh n = 4; NTS + MMF n = 4. Unpaired t-test was used to determine statistical significance. Significance was reached at *p* < 0.05. Data are presented as mean ± SD. NTS: nephrotoxic serum, MMF: mycophenolate mofetil, ns: not significant, *** *p* < 0.001* *p* < 0.05.

All mice showed severe proteinuria on day 3 (Fig. 2a). The average proteinuria at this time point, before the initiation of MMF treatment, did not differ significantly between NTS + veh and NTS + MMF mice. Importantly, one-week treatment with MMF resulted in a remarkable difference in the reduction of proteinuria compared to NTS + veh group (Fig. 2b). In line with that, histologic analysis showed a significant increase of the number of crescentic glomeruli in NTS + veh group, whereas NTS + MMF animals presented with significant less glomerular crescent (Fig. 2c). Effective exposure to the MMF administration was confirmed by TDM, the average eMPA-AUC_0–24 h_ was 25.6 ± 13.9 mg*h/L.

**Figure 3:**
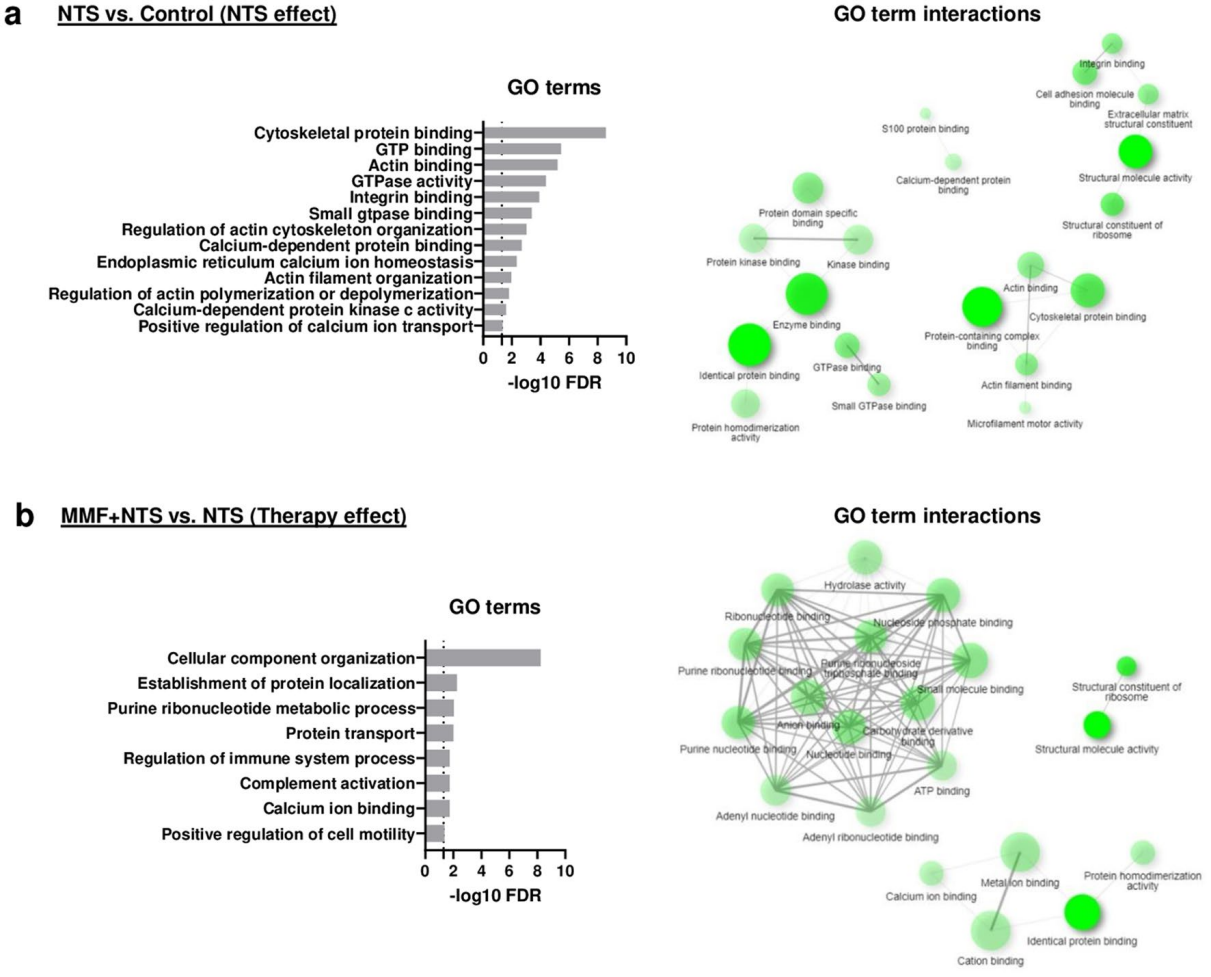
Functional analysis of significantly (*p* < 0.05) and relevantly (log2 fold change >|0.58|) altered proteins from the glomeruli proteome analysed using the ShinyGo Gene Ontology (GO) Enrichment Analysis. Panel (**a**) shows the comparison between NTS and control mice (NTS effect). Panel (**b**) shows the comparison between NTS + MMF and NTS + veh mice (therapy effect). On the left: the lollipop graphic of altered GO Molecular Functions; on the right: network analysis of the altered GO Molecular Functions. Controls n = 4; NTS + veh n = 4; NTS + MMF n = 6; NTS: nephrotoxic serum; MMF: mycophenolate mofetil.

**Figure 4:**
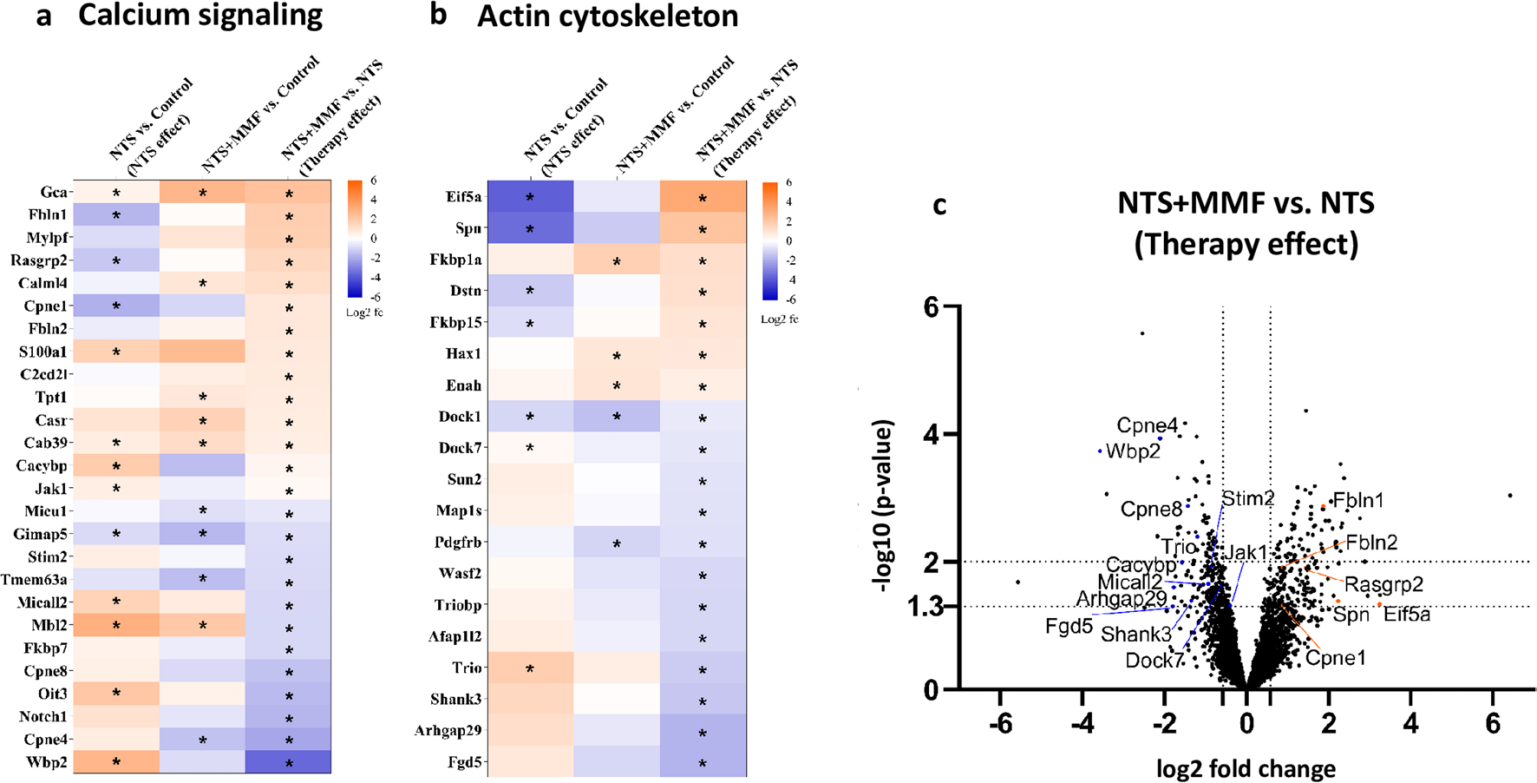
Panel (**a**) depicts the heat map of proteins related to the pathway “Calcium signaling”, while Panel (**b**) shows the heat map of proteins related to the pathway “Actin cytoskeleton”. The proteins that displayed a significant different expression level in the NTS + veh group in comparison to controls were considered as “NTS effect”. While the proteins that displayed a significant different expression level in the NTS + MMF group in comparison to the NTS + veh group were considered as “therapy effect”. An unchanged expression level in the middle row (NTS + MMF vs. control) indicated a therapy-mediated reversion to the normal state suggesting that MMF effectively restored specific cellular pathways. The *p* value < 0.05 is marked with an asterisk *; color mapping shows the fold change of each protein: Blue represents down-regulation, while orange represents up-regulation. Panel (**c**) highlights interesting proteins on a volcano plot from the comparison NTS + veh vs. NTS + MMF. Controls n = 4; NTS + veh n = 4; NTS + MMF n = 6; NTS: nephrotoxic serum, MMF: mycophenolate mofetil.

To elucidate the molecular mechanisms during MMF therapy in NTN, we performed proteomics of glomeruli from healthy controls, NTS + veh and NTS + MMF mice. In total, 3.650 glomerular proteins were detected. Out of them, 173 proteins were down-regulated and 192 proteins were up-regulated significantly (*p* value of < 0.05) and relevantly (linear fold change of > 1.5) when comparing NTS + MMF with NTS groups. To clarify molecular functions and biological processes affected by MMF-regulated proteins, we performed Gene Ontology (GO) analyses. Figure 3 depicts a selection of significant GO terms. From the enrichment analysis, we could outline two major groups of terms that were of particular interest: Ca^2+^ signaling and regulation of actin cytoskeleton (Fig. 3a, b). Of the numerous identified proteins with a *p* value < 0.05 (Supp. Table 3), we analyzed the ones related to Ca^2+^ signaling and/or actin cytoskeleton regulation displayed as a heat map (Fig. 4a, b). Additionally, the distribution of the most relevant proteins for Ca^2+^ signaling and actin cytoskeleton regulation are shown in volcano plot (Fig. 4c). For Ca^2+^signaling, two proteins attracted our interest (Supp. Table 3). Wbp2, which leads to Ca^2+^ influx from the ER into the cytoplasm. In addition, Stim2, which is responsible for the Ca^2+^ influx from the extracellular into the intracellular fluid. Both were significantly down-regulated in NTS + MMF mice compared to NTS + veh group. In line with that, we detected significant changes in the expression of Ca^2+^-dependent proteins such as Cpne1, Cpne4, Cpne8, Fbln1, Fbln2 and Rasgrp2 (Supp. Table 3). Another group of altered proteins was involved in the regulation of the actin cytoskeleton. The protein expression level of several positive regulators for the Rac1 such as Micall2, Dock7, Trio and Fgd5 were highly down-regulated in NTS + MMF vs. NTS + veh group (Supp. Table 3). The significant reduction in the expression level of the down-stream protein Wasf2 also points in this direction (Supp. Table 3). Additionally, we found the expression level of Arhgap29, a positive regulator for RhoA, highly up-regulated (Supp. Table 3). It was in line with the increased protein level of Spn and Eif5a, which are known to promote stress fiber formation (Supp. Table 3). Finally, we observed a decreased expression level of the lamellipodia inducing Shank3 (Supp. Table 3).

The effective exposure to MPA is indirectly confirmed by the compensatory up-regulation of IMPDH2, the main target of MPA (*see PRIDE repository*).

**Figure 5:**
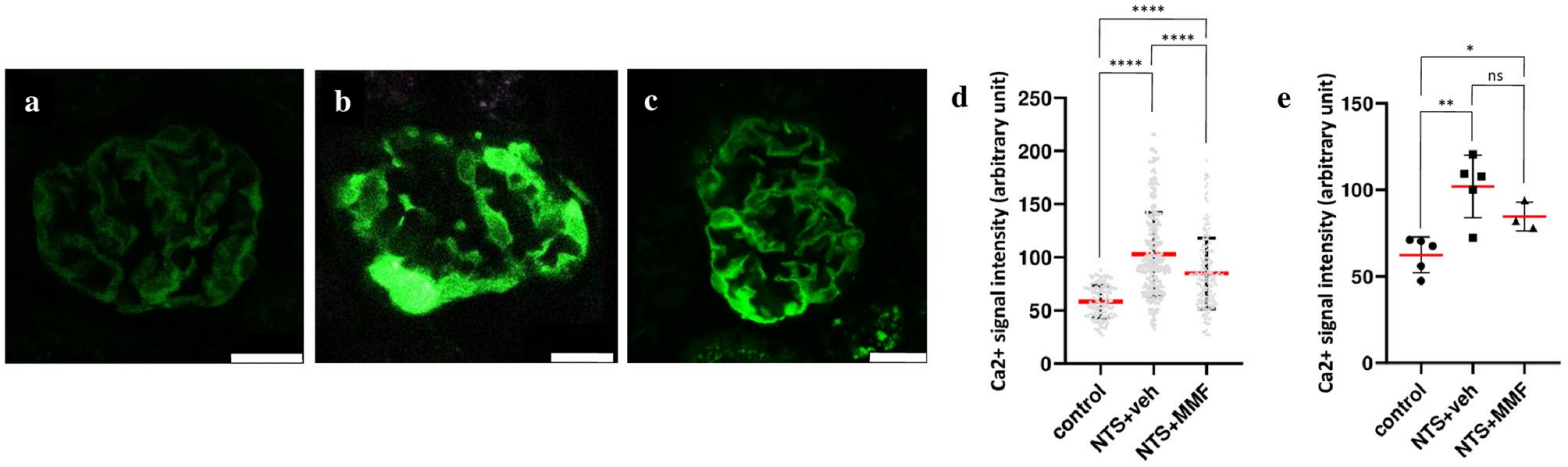
Representative images of a glomerulus of a Pod:cre GCaMP3 (green) mouse depict calcium signal in the nephrotoxic serum nephritis model. Panel (**a**) shows low signal intensity of physiologic [Ca^2+^]_i_ with only minor variations between different glomeruli. Panel (**b**) demonstrates the pronounced signal intensity of the NTS induced Ca^2+^ response. Panel (**c**) presents a moderate, but significant decrease in signal intensity indicating lower total [Ca^2+^]_i_ in podocytes after MMF treatment. Panel (**d**) shows the quantification of Ca^2+^ signal, where values correspond to the average fluorescent intensity of one glomerulus and Panel (**e**) shows the quantification of Ca^2+^ signal, where values correspond to the average fluorescent intensity of one mouse. Scale bar: 20 µm. controls n = 5; NTS + veh n = 5, NTS + MMF n = 3; At least twenty glomeruli per slice and two slices per animal were used for the quantification. One-way ANOVA and a two-sample t-test were used to determine statistical significance. Significance was reached at *p* < 0.05. Data are presented as mean ± SD. **** *p* < 0.0001, ** *p* < 0.01 * *p* < 0.05; NTS: nephrotoxic serum, MMF: mycophenolate mofetil, veh: vehicle; ns: not significant.

To quantitatively determine the [Ca^2+^]_i_, we used a transgenic mouse line with podocyte specific expression of the Ca^2+^ indicator GCaMP3 for a multiphoton imaging approach on AKS. Multiphoton imaging revealed low GCaMP3 fluorescence in healthy podocytes of controls and only minor variations between various glomeruli (Fig. 5a). Induction with NTS potently induced a pronounced increase in [Ca^2+^]_i_ in podocytes as indicated by changes in the fluorescence of GCaMP3 (Fig. 5b, d, e). Treatment with MMF resulted in a moderate, but significant decrease in podocyte [Ca^2+^]_i_ (Fig. 5c, d, e).

**Figure 6:**
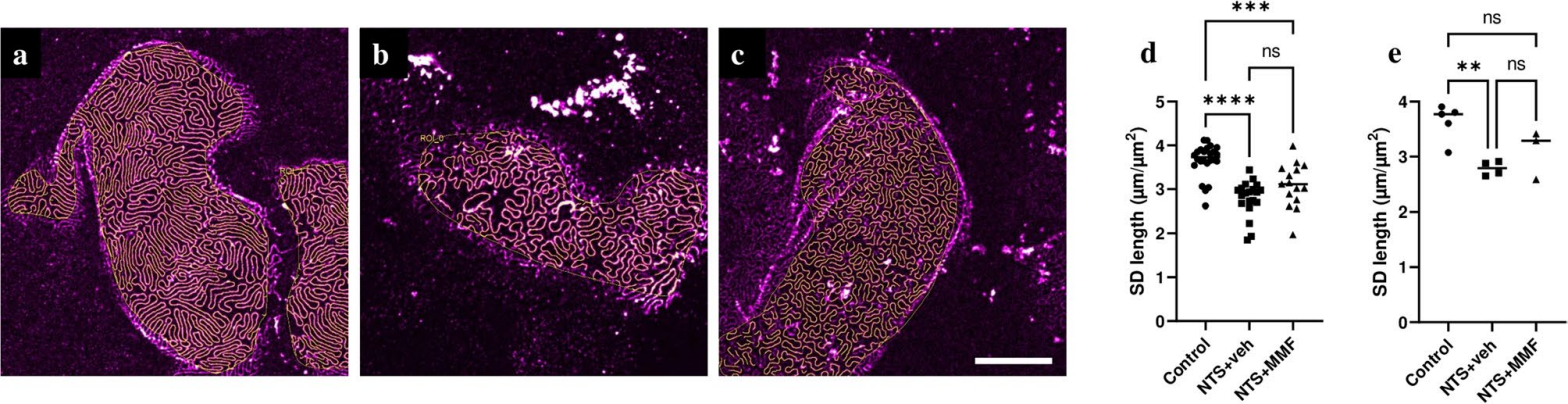
STED microscopy of podocytes foot process architecture in nephrotoxic nephritis model. The result of semiautomatic SD segmentation together with the manually assigned ROI shown as an overlay in all images (yellow). Panel (**a**) shows healthy, regular staining pattern of foot processes outlined by the nephrin staining. Panel (**b**) illustrates morphological changes after NTS induction, where the nephrin staining pattern appears less dense, resulting in significant shortening of the SD length. Panel (**c**) demonstrates a mild trend to normalization of the ultrastructural alterations of foot processes. Panel (**d**) shows quantification of the SD length, where values correspond to the average of one glomerulus and Panel (**e**) shows quantification of the SD length, where values correspond to the average of one mouse. Scale bar: 5 µm; controls n = 5, NTS + veh n = 4, NTS + MMF n = 3; At least five images per mouse were used for the quantification. One-way ANOVA and a two-sample t-test were used to determine statistical significance. Data are presented as mean ± SD. **** *p* < 0.0001, *** *p* < 0.001; NTS: nephrotoxic serum, MMF: mycophenolate mofetil, veh: vehicle; ns: not significant.

In order to assess ultrastructural changes of foot process morphology, we used STED super resolution imaging of glomeruli. We quantified morphologic alterations by measuring the SD length visualized by nephrin immunostaining. Control animals showed dense SD patterns of healthy, elongated foot processes (FPs) *(*Fig. 6a). In contrast, podocytes injured by NTS exhibited morphological changes such as reduction in the number and widening of the FPs as well as a significant shortening of the SD length (Fig. 6b). Importantly, MMF therapy improved these morphological changes (Fig. 6c). Although Fig. 6d, e demonstrate a clear trend, no significant difference was found between NTS + MMF and NTS + veh mice. Of note, a healthy SD pattern indicates an intact actin cytoskeleton as demonstrated by a synaptopodin co-staining, whereas widening and shortening of foot processes are accompanied by disruption of the actin stress fibers (Supp. Fig. 1).

## Discussion

This is the first study to investigate the molecular effects of MMF at the level of the glomerular proteome in NTN model with a clinically relevant treatment protocol. We found that MMF ameliorated proteinuria and glomerular crescent formation within one week. We identified significantly changed protein expression related to Ca^2+^ signaling and actin cytoskeleton regulation, which was further confirmed by direct [Ca^2+^]_i_ imaging in podocytes showing an improvement after MMF treatment. This functional change resulted in a tendency towards the structural stabilization of podocyte FPs. Thus, our data indicate that MMF has a beneficial effect on NTN partially by the restoration of the Ca^2+^- actin cytoskeleton axis in podocytes.

The NTN model possesses several advantages of an immunologically triggered pathomechanism and a rapid induction phase. This enabled us to investigate young mice at the age of four and a half weeks, when the kidneys are still growing, in order to model the dynamic immunological changes in our pediatric population. Additionally, our induction and treatment protocol mirrors the clinical reality of childhood glomerulopathies, as the start of the therapy was not preventive, but held for three days after disease induction to a time point, where all experimental animals exhibited proteinuria. In order to monitor the applied doses, we established TDM via MPA-AUC_0–24 h_, which is the first time implemented in a mouse model. By performing TDM of MPA, we were able to show effective exposure to MPA. We know from previous clinical studies that an estimated MPA-AUC reflects MPA exposure more reliably and correlates better with the clinical outcome than MPA-C_0_ values do^28–30^.

**Figure 7:**
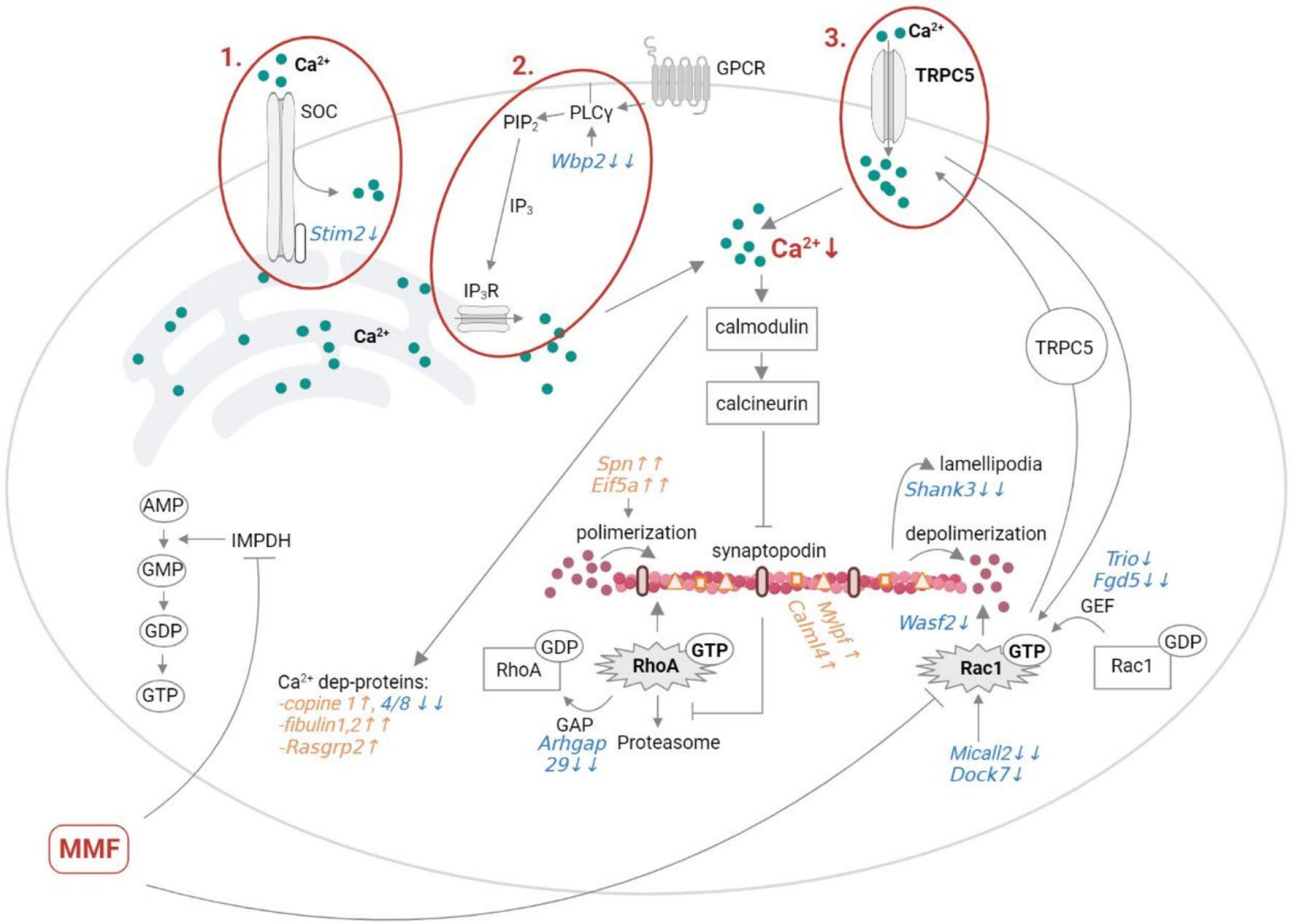
Molecular changes in podocytes after MMF treatment in nephrotoxic serum nephritis. When comparing NTS + veh vs. NTS + MMF, we identified significantly changed protein expressions related to Ca^2+^signaling reducing overall the Ca^2+^influx into cytoplasm. This partially normalized [Ca^2+^]_i_ can influence the expression of Ca^2+^-dependent proteins and prevent the cleavage of synaptopodin and RhoA. Additionally, MMF treatment leads to expressional changes of several regulatory proteins of Rac1, acting in the direction of Rac1 inactivation. While it promotes the activation of RhoA and the expression of other proteins leading overall to the preservation of stress fibers and stationary phenotype of podocytes. Up-regulation is colored in orange, down-regulation in blue. Red circles highlight the signaling pathways (1–3), which are influenced by MMF treatment and can lead to the restoration of [Ca^2+^]_i._ Created with BioRender.com.

We sought to elucidate the differential molecular signaling pathways that may account for renoprotection conferred by the administration of MMF therapy in our NTN model. Comparison of proteomics profiles showed that terms of “Ca^2+^ signal” and “actin cytoskeleton regulation” were enriched after MMF treatment, suggesting their role in improving the kidney injury in NTN. Ca^2+^dependent remodeling of the actin cytoskeleton is a dynamic process that enables adaptation of podocytes under physiologic and adverse conditions. Consequently, dysfunction of this dynamic process can induce detachment from the basement membrane and podocyte loss. This dynamic process is regulated by modulating the activity of members of the Rho family of small GTPases. RhoA promotes the formation of stress fibers to generate a stable foot processes. In contrast, Rac1 promotes disease-associated cell motility^31,32^. Intriguingly, some of the immunosuppressive drugs have been shown to preserve the actin cytoskeleton of podocytes^10,33,34^. In line with these data, we found that MMF has a direct effect on the proteome of glomerular cells (Fig. 7): MMF diminished the protein expression of Stim2, which regulates the Ca^2+^ influx through the store-operated Ca^2+^-channels^35,36^. Additionally, MMF lowered the protein expression of Wbp2, which may influence the Ca^2+^ release from the ER^37^. Similar to earlier published proteomic analyses of glomeruli^38^, we were unable to quantify TRPC channels, supposedly due to their very low abundance. Nevertheless, it was previously shown that the membrane incorporation of TRPC5 requires activated Rac1^39–41^, which was in turn down-regulated by MMF in a murine lupus model^10^. This is supported by our proteomics data where the expression of several proteins (Trio, Fgd5, Micall2, Dock7), which activate Rac1, was diminished. In addition, Wasf2, a down-stream target of the Rac1 pathway, was down-regulated, indicating a lower activation of Rac1 in our model, too. Subsequently, we assume that the feedback loop between Rac1-TRPC5-Ca^2+^influx-Rac1^39^ is at least partially disrupted by MMF. Altogether, MMF targets several pathways reducing the overall Ca^2+^influx into the cytoplasm, which further impacts down-stream signaling pathways. Accordingly, MMF may influence cell functions by changing the expression of Ca^2+^-dependent proteins: e.g. activation of integrin through Rasgrp2, stabilization of the extracellular matrix via fibulins and diverse cascades via copinins. In addition, potential blocking of the calmodulin-calcineurin axis may prevent the cleavage of synaptopodin and RhoA as described earlier^42^. Furthermore, MMF treatment resulted in expressional changes of several regulatory proteins of Rac1, all acting in the direction of Rac1 inactivation. While the decreased expression of Arhgap29 promoted the activation of RhoA. Thus, our dataset suggests that MMF mediates a reciprocal regulation of RhoA and Rac1 pathways leading to the overall preservation of stress fibers and stationary phenotype of podocytes. This was further indicated by the alterations of proteins such as Spn, Eif5a, Mylpf, Calml4 and Shank3. Our proteomics analysis revealed that MMF could beneficially influence the Ca^2+^ signal and facilitate the formation of stress fibers. Although these data originate from cells of the whole glomeruli, but are very likely influenced by podocytes, as they heavily rely on an intact Ca^2+^-actin cytoskeleton axis^14^. It is also in line with our previous in vitro experiments^11^, which we performed to exclude the impact of immune cells and study MPA’s direct effect on podocytes. Doing so, our RNA sequencing of an MPA treated podocyte cell line revealed an accumulation of terms including “calcium channel activity”, “calcium ion transport” “actin cytoskeleton” and “regulation of small GTPase mediated signal transduction*”.* This leads potentially to normalization of [Ca^2+^]_i_ in podocytes and to amelioration of the disorganized actin cytoskeleton demonstrated also by immunofluorescence staining^11^.

To validate our proteomics data on Ca^2+^-related proteins in podocytes, we performed multiphoton microscopy on AKS of mice expressing the [Ca^2+^]_i_ sensor protein GCaMP3 specifically in podocytes^13,15^. The use of AKS enables imaging of many glomeruli in three dimensions reducing the selection bias. This is particularly advantageous in our NTN model due to heterogeneous population of glomerular lesions. The rise in [Ca^2+^]_i_ is an early event in podocyte injury^12^ and the excess [Ca^2+^]_i_ can lead to acute reorganization of the actin cytoskeleton or even cytoskeletal collapse^13–16^. Therefore, the tight control of the [Ca^2+^]_i_ is essential in these cells. We also detected significant elevation of [Ca^2+^]_i_ in our disease model which may contribute to the activation of pathologic signaling pathways in NTN. Importantly, MMF therapy reduced the elevated [Ca^2+^]_i_ in podocytes. In summary, our proteomics and imaging data were concordant: The decreased expression of Ca^2+^-channel triggering proteins and the indicated decreased activation rate of Rac1 in MMF treated mice, were confirmed by directly visualizing the reduced podocyte [Ca^2+^]_i_ after the treatment with MMF.

To validate the proteomics data related to actin cytoskeleton regulation and their consequence on the podocytes’ FP structure, we applied the STED microscopy imaging of FPs, which enables us to stain for proteins of the filtration barrier^26,27^. In the present study, we detected significantly reduced SD length in the NTN group, indicating a dysfunction of the glomerular filtration barrier. Importantly, MMF therapy could slightly improve the structure of FPs. The lack of statistical significance might be explained by the short exposure to MMF or the relative low number of analyzed glomeruli in the heterogeneous NTN model. In summary, our STED data confirmed the proteomics data by showing a tendency to the restoration of the FP’ structure and suggesting the stabilization of the actin cytoskeleton in the MMF group.

We acknowledge that our study also comes with several limitations: (1) We found that one-week MMF treatment improved proteinuria, however, not significantly. Thus, MMF has the potential to beneficially influence signal pathways and histological changes in NTN, but either the duration of therapy was too short in our reversible model or MMF alone has only limited efficacy to induce a complete remission in proteinuria resulting from the interplay of a plethora of down-stream signaling pathways. (2) We were also not able to resolve microdomain Ca^2+^ signaling at the cell membrane using our cytosolic calcium indicator, as [Ca^2+^]_i_ imaging via multiphoton microscopy serves rather as an indirect readout of all Ca^2+^-channels showing the total [Ca^2+^]_i_. However, this increase of [Ca^2+^]_i_ in the whole podocyte reflects a breakdown of mechanisms to control microdomain Ca^2+^ signaling. In our opinion, this might reflect the general condition of the podocytes even better. (3) Since our study focused on podocytes, we cannot rule out, that MMF simultaneously improved the function of injured mesangial cells and glomerular endothelial cells, thus, the observed effects in podocytes might have also been influenced by altered glomerular cellular cross-talk. (4) Finally, it was challenging to clearly differentiate the systemic effects of immunosuppression by MMF vs. its direct effects on the glomerular cells, as they are inseparably connected in an in vivo model, though this resembles the situation in our patients. Nevertheless, we believe that the application of targeted [Ca^2+^]_i_ imaging and STED studies of podocytes helped to understand the specific direct effect in podocytes.

In summary, we identified signaling pathways in glomeruli regulated by MPA and revealed new mechanistic understanding of how MMF reduces proteinuria in a podocyte specific manner, beyond its systemic immunosuppressive effect: MMF contributes to the normalization of [Ca^2+^]_i_ and ameliorates the disorganized actin cytoskeleton in podocytes thus improving the glomerular barrier function. In conclusion, our study provides the pathophysiological rationale for utilizing MMF’s direct effects on podocytes in addition to its immunosuppressive action in proteinuric glomerulopathies to obtain a more effective treatment with the least possible side effects.

### Supplementary Information

Supplementary Information 1.

Supplementary Information 2.

## Data Availability

The mass spectrometry proteomics data have been deposited to the ProteomeXchange Consortium via the PRIDE^43^ partner repository with the dataset identifier PXD039915.

## Abbreviations

AKS: Acute kidney slices
Ca^2+^: Calcium ion
[Ca^2+^]_i_: Intracellular Ca^2+^ concentration
FP: Foot process
GO: Gene ontology
IMPDH: Inosin-5′-monophosphate dehydrogenase
KHB: Krebs–Henseleit-buffer
MMF: Mycophenolate mofetil
MPA: Mycophenolic acid
NTN: Nephrotoxic serum nephritis
NTS: Nephrotoxic serum
PAS: Periodic acid–schiff
PBS: Phosphate-buffered saline
PFA: Paraformaldehyde
Rac1: Ras-related C3 botulinum toxin substrate 1
RhoA: Ras homolog gene family, member A
SD: Slit diaphragm
STED: Stimulated emission depletion
TDM: Therapeutic drug monitoring
