# Supplementary figures and images for: The effect of mycophenolate mofetil on podocytes in nephrotoxic serum nephritis
Source: Sci Rep. 2023 Aug 29;13:14167. doi: 10.1038/s41598-023-41222-1 (PMC10465485; doi:10.1038/s41598-023-41222-1)

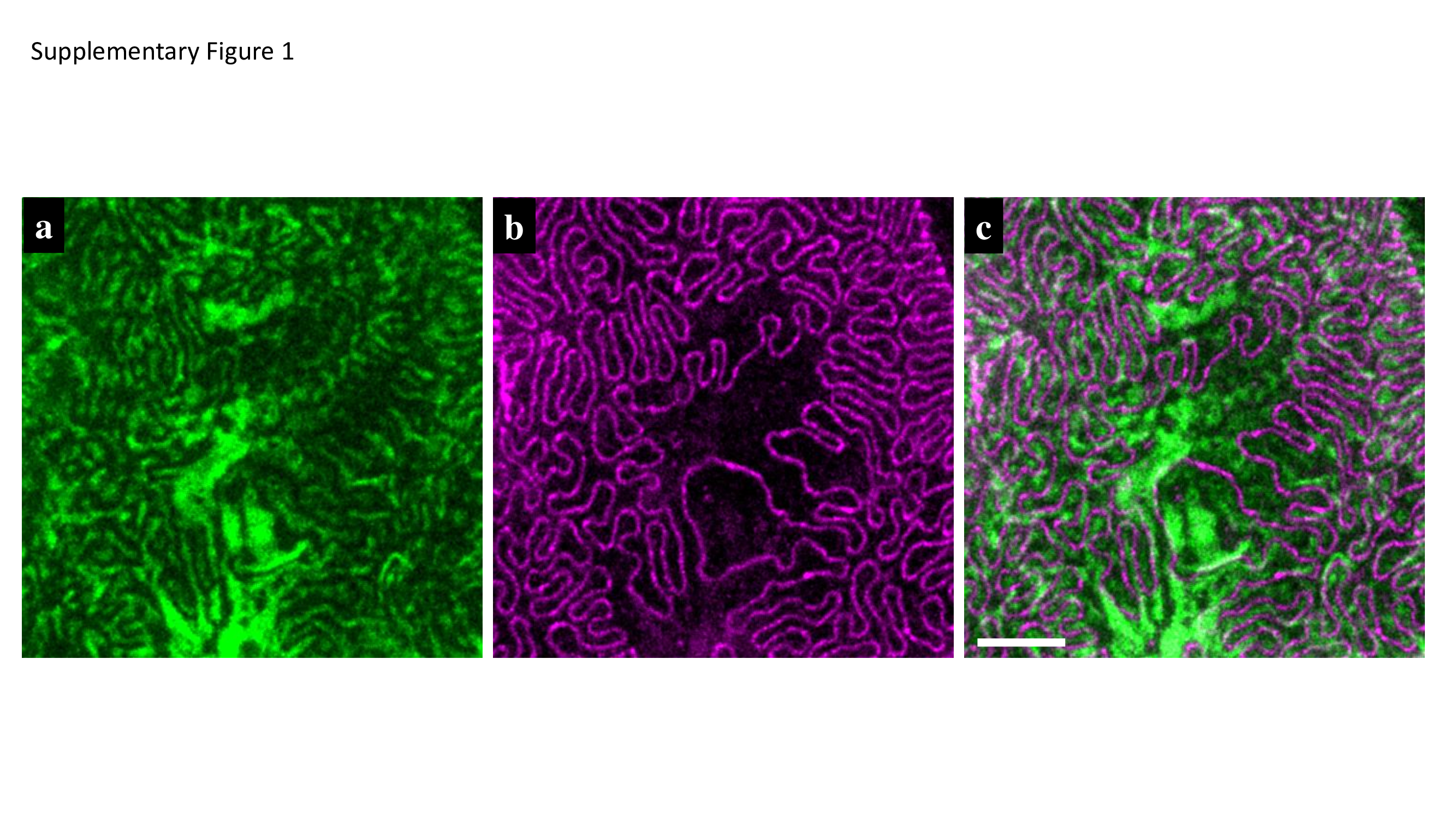

Supplement: Supplementary file 1 — Supplementary Information 1. [file 41598_2023_41222_MOESM1_ESM.tiff]
