# Supplementary material for: The effect of mycophenolate mofetil on podocytes in nephrotoxic serum nephritis
Source: Sci Rep. 2023 Aug 29;13:14167. doi: 10.1038/s41598-023-41222-1 (PMC10465485; doi:10.1038/s41598-023-41222-1)
Supplement: Supplementary file 2 — Supplementary Information 2. [file 41598_2023_41222_MOESM2_ESM.docx]

***Supplementary Figure 1:*** *Representative image of a glomerulus after induction with nephrotoxic serum. The co-staining of actin-associated synaptopodin (green) and the slit diaphragm component nephrin (purple) shows that the intact nephrin localization highly influences the accompanying actin-associated synaptopodin signal and vice versa****.*** *Scale bar: 2 µm.*

***Supplementary Table 1*** *shows the primers used and the resultingfragment sizes detected in gel electrophoresis. Panel a) genotyping for Cre recombinase with beta-globulin as internal control; Panel b) genotyping for GCaMP3 in the Rosa26 locus; wt: wilde type, bp: base pair*

***Supplementary Table 2*** *Overview of the antibodies used for STED imaging*

***Supplementary Table 3*** *Table of significantly and relevantly altered glomerular proteins (p-value p<0.05; log2 fold change >|0.58|) between the comparison NTS+MMF and NTS (Therapy effect). Red marked p-values are also significant after Bonferroni adjustment. The log 2 fold changes are color mapped. Upregulation is mapped in orange, downregulation in blue.*

**Supplementary Table 1**

**a)**

| Beta-globin forward | TGC TCA CAC AGG ATA GAG AGG GCA GG |
| --- | --- |
| Beta-globin reverse | GGC TGT CCA AGT GAT TCA GGC CAT CG |
| Creforward | GCA TAA CCA GTG AAA CAG CAT TGC TG |
| Cre reverse | GGA CAT GTT CAG GGA TCG CCA GGC G |
| Fragment size of a wt mouse | 494 bp |
| Fragment size of a transgenic mouse | 269 bp + 494 bp |

**b)**

| oIMR7318 | CTC TGC TGC CTC CTG GCT TCT |
| --- | --- |
| oIMR7319 | CGA GGC GGA TCA CAA GCA ATA |
| oIMR7320 | TCA ATG GGC GGG GGT CGT T |
| Fragment size of a wt mouse | 330 bp |
| Fragment size of a transgenic mouse | 250 bp |

**Supplementary Table 2**

| Antibody | catalog number | dilution | temperatur | lenght of incubation | distributor |
| --- | --- | --- | --- | --- | --- |
|  |  |  |  |  |  |
| guinea pig anti-nephrin | 20R-NP002 | 1:100 | 37 °C | 24h | Fitzgerald, Poggensee, Germany |
| donkey anti–guinea pig | 706–005–148 | 1:50 | 37 °C | 24h | Jackson Immuno, Ely, United Kingdom |
| conjugated to STAR635P | 7679 |  |  |  | Sigma-Aldrich, Taufkirchen, Germany |
|  |  |  |  |  |  |
| rabbit anti-synaptopodin | HPA034631 | 1:100 | 37 °C | 24h | Sigma-Aldrich, Taufkirchen, Germany |
| donkey anti-rabbit | A16037 | 1:100 | 37 °C | 24h | Thermo Fisher Scientific, Bremen, Germany |
| conjugated to Atto594 | 8741 |  |  |  | Sigma-Aldrich, Taufkirchen, Germany |

**Supplementary Table 3**

| Relevantly altered glomerular proteins | | -log 10 p-value | | | log 2 fold change | | |
| --- | --- | --- | --- | --- | --- | --- | --- |
| *Gene name* | Protein name | NTS vs. Control  (NTS effect) | NTS+MMF vs. Control | NTS+MMF vs. NTS  (Therapy effect) | NTS vs. Control  (NTS effect) | NTS+MMF vs. Control | NTS+MMF vs. NTS  (Therapy effect) |
| Cse1l | Exportin-2 | 0.025 | 0.738 | 0.021 | 6.37 | 0.80 | -5.58 |
| Wbp2 | WW domain-bindingprotein 2 | 0.001 | 0.052 | <0.001 | 2.73 | -0.84 | -3.57 |
| F13a1 | Coagulation factor XIII A chain | <0.001 | 0.172 | 0.001 | 4.78 | 1.37 | -3.41 |
| Senp7 | Sentrin-specificprotease 7 | 0.023 | 0.001 | <0.001 | 0.98 | -1.55 | -2.54 |
| Gpatch8 | G patch domain-containing protein 8 | 0.051 | 0.050 | 0.004 | 0.92 | -1.25 | -2.17 |
| Cpne4 | Copine-4 | 0.124 | 0.004 | <0.001 | 0.66 | -1.45 | -2.11 |
| Fgd5 | FYVE, RhoGEF and PH domain-containing protein 5 | 0.358 | 0.307 | 0.050 | 0.85 | -0.95 | -1.80 |
| Arhgap29 | RhoGTPase-activatingprotein 29 | 0.222 | 0.522 | 0.025 | 1.20 | -0.58 | -1.78 |
| Dpf2 | Zincfingerprotein ubi-d4 | 0.218 | 0.173 | 0.036 | 0.96 | -0.80 | -1.76 |
| Pds5a | Sister chromatid cohesion protein PDS5 homolog A | 0.041 | 0.947 | 0.008 | 1.69 | -0.05 | -1.74 |
| Notch1 | Neurogeniclocusnotch homolog protein 1 | 0.112 | 0.188 | 0.017 | 1.10 | -0.63 | -1.73 |
| Ighm | Immunoglobulin heavy constantmu | 0.005 | 0.494 | 0.003 | 2.09 | 0.40 | -1.68 |
| Igkc | Immunoglobulinkappaconstant | <0.001 | 0.006 | <0.001 | 3.15 | 1.47 | -1.68 |
| Mpp2 | MAGUK p55 subfamilymember 2 | 0.290 | 0.052 | 0.004 | 0.58 | -1.10 | -1.68 |
| Oit3 | Oncoprotein-inducedtranscript 3 protein | <0.001 | 0.150 | <0.001 | 2.10 | 0.47 | -1.63 |
| Wdr37 | WD repeat-containingprotein 37 | 0.840 | 0.008 | 0.003 | -0.12 | -1.75 | -1.63 |
| Eno3 | Beta-enolase | 0.081 | 0.596 | 0.024 | 1.31 | -0.29 | -1.60 |
| Sptb | Spectrinbetachain, erythrocytic | 0.417 | 0.188 | 0.041 | 0.67 | -0.91 | -1.58 |
| Cacybp | Calcyclin-bindingprotein | 0.007 | 0.544 | 0.010 | 1.92 | 0.35 | -1.57 |
| Ushbp1 | Harmonin-bindingprotein USHBP1 | 0.006 | 0.088 | <0.001 | 0.97 | -0.53 | -1.50 |
| Oasl2 | 2'-5'-oligoadenylate synthase-like protein 2 | 0.445 | 0.089 | 0.048 | 0.53 | -0.97 | -1.50 |
| Cd5l | CD5 antigen-like | 0.002 | 0.075 | 0.016 | 2.76 | 1.29 | -1.47 |
| Cpne8 | Copine-8 | 0.232 | 0.059 | 0.001 | 0.55 | -0.89 | -1.44 |
| Phf6 | PHD fingerprotein 6 | 0.086 | 0.162 | 0.014 | 0.90 | -0.52 | -1.43 |
| Larp4 | La-relatedprotein 4 | <0.001 | 0.034 | 0.001 | 2.38 | 0.97 | -1.41 |
| Bag5 | BAG family molecular chaperone regulator 5 | 0.079 | 0.266 | 0.003 | 0.91 | -0.50 | -1.41 |
| Shank3 | SH3 and multiple ankyrin repeat domains protein 3 | 0.061 | 0.892 | 0.041 | 1.44 | 0.09 | -1.35 |
| Nova1 | RNA-bindingprotein Nova-1 | 0.118 | 0.287 | 0.017 | 0.85 | -0.50 | -1.35 |
| Babam1 | BRISC and BRCA1-A complex member 1 | 0.017 | 0.672 | 0.011 | 1.54 | 0.23 | -1.31 |
| Hmgb3 | High mobility group protein B3 | 0.002 | 0.009 | 0.036 | 2.64 | 1.34 | -1.30 |
| Znf592 | Zincfingerprotein 592 | 0.352 | 0.046 | 0.022 | 0.45 | -0.84 | -1.29 |
| Ddrgk1 | DDRGK domain-containingprotein 1 | 0.245 | 0.089 | 0.009 | 0.55 | -0.73 | -1.28 |
| Zdhhc20 | Palmitoyltransferase ZDHHC20 | 0.005 | 0.431 | 0.001 | 1.04 | -0.22 | -1.26 |
| Masp1 | Mannan-bindinglectinserineprotease 1 | <0.001 | 0.003 | 0.001 | 2.59 | 1.35 | -1.24 |
| Sash1 | SAM and SH3 domain-containing protein 1 | 0.279 | 0.019 | 0.003 | 0.37 | -0.87 | -1.24 |
| Col6a5 | Collagen alpha-5 (VI) chain | <0.001 | 0.007 | 0.001 | 2.24 | 1.03 | -1.21 |
| Icam2 | Intercellularadhesionmolecule 2 | 0.001 | 0.640 | <0.001 | 1.10 | -0.11 | -1.21 |
| Igsf3 | Immunoglobulinsuperfamilymember 3 | 0.031 | 0.741 | 0.036 | 1.34 | 0.12 | -1.21 |
| Trio | Triple functionaldomainprotein | 0.001 | 0.158 | 0.004 | 1.83 | 0.63 | -1.20 |
| Rpl29 | 60S ribosomalprotein L29 | 0.041 | 0.776 | 0.007 | 1.07 | -0.13 | -1.20 |
| Gng5 | Guanine nucleotide-binding protein G | 0.023 | 0.726 | 0.027 | 1.30 | 0.12 | -1.18 |
| Masp2 | Mannan-bindinglectinserineprotease 2 | 0.002 | 0.029 | 0.024 | 2.86 | 1.72 | -1.13 |
| Serping1 | Plasma protease C1 inhibitor | 0.012 | 0.156 | 0.047 | 1.69 | 0.57 | -1.12 |
| C1sa | Complement C1s-1 subcomponent | 0.027 | 0.274 | 0.047 | 1.82 | 0.71 | -1.11 |
| Tlr4 | Toll-like receptor 4 | 0.044 | 0.590 | 0.018 | 0.88 | -0.22 | -1.10 |
| Rpl15 | 60S ribosomalprotein L15 | 0.195 | 0.002 | 0.014 | -0.63 | -1.72 | -1.09 |
| Jam2 | Junctionaladhesionmolecule B | 0.002 | 0.462 | 0.001 | 1.33 | 0.25 | -1.08 |
| Trappc11 | Trafficking protein particle complex subunit 11 | 0.052 | 0.047 | <0.001 | 0.55 | -0.52 | -1.07 |
| Reps1 | RalBP1-associated Eps domain-containing protein 1 | 0.012 | 0.897 | 0.005 | 1.02 | -0.04 | -1.06 |
| Ndc1 | Nucleoporin NDC1 | 0.006 | 0.168 | 0.030 | 1.66 | 0.60 | -1.06 |
| Fbxl20 | F-box/LRR-repeat protein 20 | 0.043 | 0.338 | 0.002 | 0.74 | -0.32 | -1.06 |
| Stk10 | Serine/threonine-protein kinase 10 | 0.088 | 0.691 | 0.022 | 0.87 | -0.18 | -1.05 |
| Tubgcp2 | Gamma-tubulincomplexcomponent 2 | 0.010 | 0.227 | 0.043 | 1.60 | 0.56 | -1.04 |
| Luc7l3 | Luc7-like protein 3 | 0.173 | <0.001 | 0.002 | -0.42 | -1.46 | -1.03 |
| Dhx8 | ATP-dependent RNA helicase DHX8 | 0.562 | 0.045 | 0.017 | 0.22 | -0.81 | -1.03 |
| Naalad2 | N-acetylated-alpha-linked acidic dipeptidase 2 | 0.021 | 0.883 | 0.012 | 1.08 | 0.06 | -1.02 |
| Cd200 | OX-2 membraneglycoprotein | 0.026 | 0.601 | 0.002 | 0.83 | -0.17 | -1.00 |
| Smc4 | Structural maintenance of chromosomes protein 4 | 0.011 | 0.891 | 0.007 | 1.03 | 0.05 | -0.98 |
| Afap1l2 | Actin filament-associated protein 1-like 2 | 0.066 | 0.253 | 0.002 | 0.62 | -0.36 | -0.98 |
| Psmb10 | Proteasomesubunitbeta type-10 | 0.185 | 0.283 | 0.015 | 0.53 | -0.45 | -0.98 |
| Col12a1 | Collagen alpha-1 (XII) chain | <0.001 | <0.001 | 0.008 | 2.82 | 1.85 | -0.97 |
| Rilpl1 | RILP-like protein 1 | 0.068 | 0.862 | 0.036 | 1.06 | 0.09 | -0.97 |
| Tnc | Tenascin | 0.006 | 0.192 | 0.032 | 1.57 | 0.60 | -0.97 |
| Pdcd10 | Programmedcelldeathprotein 10 | 0.227 | 0.006 | 0.030 | -0.53 | -1.50 | -0.97 |
| Tap2 | Antigen peptidetransporter 2 | 0.206 | 0.003 | 0.011 | -0.40 | -1.36 | -0.96 |
| Triobp | TRIO and F-actin-binding protein | 0.181 | 0.139 | 0.002 | 0.47 | -0.49 | -0.96 |
| Fkbp7 | Peptidyl-prolyl cis-trans isomerase FKBP7 | 0.221 | 0.172 | 0.016 | 0.48 | -0.47 | -0.95 |
| Sypl1 | Synaptophysin-like protein 1 | 0.039 | 0.630 | 0.010 | 0.79 | -0.16 | -0.95 |
| Mbl2 | Mannose-bindingprotein C | <0.001 | 0.002 | 0.039 | 2.95 | 2.00 | -0.95 |
| Il6st | Interleukin-6 receptorsubunitbeta | <0.001 | <0.001 | 0.013 | -2.12 | -3.06 | -0.94 |
| Micall2 | MICAL-like protein 2 | 0.003 | 0.120 | 0.022 | 1.66 | 0.72 | -0.94 |
| Srprb | Signal recognition particle receptor subunit beta | 0.724 | 0.077 | 0.014 | 0.15 | -0.78 | -0.93 |
| Cdc16 | Cell division cycle protein 16 homolog | 0.284 | 0.046 | 0.005 | 0.35 | -0.59 | -0.93 |
| Ufd1 | Ubiquitin recognition factor in ER-associated degradation protein 1 | 0.004 | 0.116 | 0.030 | 1.71 | 0.78 | -0.93 |
| Ctcf | Transcriptionalrepressor CTCF | 0.073 | 0.528 | 0.006 | 0.71 | -0.22 | -0.93 |
| Akap9 | A-kinaseanchorprotein 9 | 0.027 | 0.447 | 0.002 | 0.71 | -0.22 | -0.93 |
| Use1 | Vesicletransportprotein USE1 | 0.029 | 0.512 | 0.004 | 0.74 | -0.18 | -0.93 |
| Tom1l2 | TOM1-like protein 2 | 0.681 | <0.001 | <0.001 | -0.09 | -1.02 | -0.93 |
| Ano1 | Anoctamin-1 | 0.537 | 0.011 | 0.001 | 0.17 | -0.75 | -0.92 |
| Wasf2 | Actin-bindingprotein WASF2 | 0.610 | 0.182 | 0.028 | 0.26 | -0.66 | -0.92 |
| Usp39 | U4/U6.U5 tri-snRNP-associated protein 2 | 0.052 | 0.922 | 0.042 | 0.94 | 0.03 | -0.91 |
| Tgfbr2 | TGF-beta receptor type-2 | 0.007 | 0.498 | 0.004 | 1.13 | 0.23 | -0.90 |
| Kank4 | KN motif and ankyrin repeat domain-containing protein 4 | 0.082 | 0.546 | 0.006 | 0.69 | -0.21 | -0.90 |
| Wtap | Pre-mRNA-splicing regulator WTAP | 0.015 | 0.981 | 0.008 | 0.89 | -0.01 | -0.90 |
| Lyar | Cell growth-regulating nucleolar protein | 0.030 | 0.982 | 0.031 | 0.90 | 0.01 | -0.90 |
| Rtf1 | RNA polymerase-associated protein RTF1 homolog | 0.208 | 0.447 | 0.028 | 0.55 | -0.34 | -0.89 |
| Dennd3 | DENN domain-containingprotein 3 | 0.706 | 0.014 | 0.013 | 0.12 | -0.76 | -0.89 |
| Hacd3 | Very-long-chain | 0.362 | 0.355 | 0.044 | 0.46 | -0.43 | -0.88 |
| Abca9 | ATP-binding cassette sub-family A member 9 | 0.996 | 0.051 | 0.035 | 0.00 | -0.88 | -0.88 |
| Rftn1 | Raftlin | 0.001 | 0.057 | 0.006 | 1.58 | 0.70 | -0.88 |
| Ddx42 | ATP-dependent RNA helicase DDX42 | 0.024 | 0.550 | 0.004 | 0.71 | -0.16 | -0.88 |
| Snrnp40 | U5 small nuclear ribonucleoprotein 40 kDa protein | 0.024 | 0.426 | 0.040 | 1.18 | 0.31 | -0.88 |
| Tmem63a | CSC1-like protein 1 | 0.102 | 0.003 | 0.030 | -0.69 | -1.57 | -0.88 |
| Rps2 | 40S ribosomalprotein S2 | 0.758 | 0.036 | 0.012 | 0.11 | -0.76 | -0.88 |
| Arl8b | ADP-ribosylation factor-like protein 8B | 0.792 | 0.030 | 0.035 | -0.11 | -0.98 | -0.87 |
| Tmtc3 | Protein O-mannosyl-transferase TMTC3 | 0.070 | 0.941 | 0.037 | 0.89 | 0.03 | -0.87 |
| Pip4p1 | Type 1 phosphatidylinositol 4,5-bisphosphate 4-phosphatase | 0.017 | NA | 0.017 | 0.87 | 0.00 | -0.87 |
| Irag1 | Inositol 1,4,5-triphosphate receptorassociated 1 | 0.386 | 0.106 | 0.006 | 0.30 | -0.56 | -0.86 |
| Kank3 | KN motif and ankyrin repeat domain-containing protein 3 | 0.141 | 0.441 | 0.025 | 0.58 | -0.27 | -0.85 |
| Stim2 | Stromalinteractionmolecule 2 | 0.128 | 0.552 | 0.012 | 0.63 | -0.22 | -0.85 |
| Med14 | Mediator of RNA polymerase II transcription subunit 14 | 0.034 | 0.765 | 0.036 | 0.97 | 0.13 | -0.85 |
| Map7d1 | MAP7 domain-containingprotein 1 | 0.013 | 0.463 | 0.033 | 1.11 | 0.27 | -0.84 |
| Acap2 | Arf-GAP with coiled-coil, ANK repeat and PH domain-containing protein 2 | 0.077 | 0.351 | 0.003 | 0.57 | -0.27 | -0.84 |
| Gnl2 | Nucleolar GTP-bindingprotein 2 | 0.005 | 0.167 | 0.020 | 1.40 | 0.57 | -0.84 |
| Birc6 | Baculoviral IAP repeat-containing protein 6 | 0.471 | 0.083 | 0.018 | 0.25 | -0.58 | -0.83 |
| Baz1a | Bromodomain adjacent to zinc finger domain protein 1A | 0.008 | 0.162 | 0.036 | 1.34 | 0.51 | -0.83 |
| She | SH2 domain-containing adapter protein E | <0.001 | 0.009 | 0.005 | 1.75 | 0.93 | -0.83 |
| Caprin1 | Caprin-1 | 0.006 | 0.343 | 0.014 | 1.14 | 0.31 | -0.83 |
| Tap1 | Antigen peptidetransporter 1 | 0.175 | 0.413 | 0.018 | 0.52 | -0.30 | -0.82 |
| Papln | Papilin | 0.004 | 0.049 | 0.044 | 2.03 | 1.21 | -0.82 |
| Slc35b2 | Adenosine 3'-phospho 5'-phosphosulfate transporter 1 | 0.003 | 0.136 | 0.016 | 1.40 | 0.58 | -0.82 |
| C1qb | Complement C1q subcomponent subunit B | <0.001 | 0.001 | 0.025 | 2.28 | 1.47 | -0.82 |
| Golga7 | Golginsubfamily A member 7 | 0.202 | 0.320 | 0.011 | 0.47 | -0.35 | -0.81 |
| Sipa1 | Signal-induced proliferation-associated protein 1 | 0.484 | 0.005 | 0.005 | -0.23 | -1.04 | -0.81 |
| Kif13a | Kinesin-like protein KIF13A | 0.003 | 0.183 | 0.039 | 1.35 | 0.55 | -0.80 |
| Gimap5 | GTPase IMAP familymember 5 | 0.057 | 0.002 | 0.039 | -0.89 | -1.69 | -0.80 |
| Ccn2 | CCN familymember 2 | 0.788 | 0.110 | 0.028 | 0.11 | -0.69 | -0.80 |
| Thoc6 | THO complexsubunit 6 homolog | 0.007 | <0.001 | 0.015 | -1.03 | -1.83 | -0.80 |
| Bst2 | Bonemarrowstromalantigen 2 | 0.005 | 0.626 | 0.004 | 0.92 | 0.13 | -0.80 |
| Vps8 | Vacuolar protein sorting-associated protein 8 homolog | 0.067 | 0.630 | 0.008 | 0.64 | -0.15 | -0.79 |
| Rpl13 | 60S ribosomalprotein L13 | 0.534 | 0.104 | 0.029 | 0.22 | -0.56 | -0.78 |
| Golga3 | Golginsubfamily A member 3 | 0.300 | 0.257 | 0.025 | 0.38 | -0.39 | -0.77 |
| Nelfb | Negative elongationfactor B | 0.043 | 0.667 | 0.044 | 0.95 | 0.18 | -0.77 |
| Cbx3 | Chromoboxprotein homolog 3 | 0.178 | 0.188 | 0.013 | 0.40 | -0.37 | -0.77 |
| S1pr3 | Sphingosine 1-phosphate receptor 3 | 0.110 | 0.630 | 0.045 | 0.58 | -0.19 | -0.76 |
| Setd1a | Histone-lysine N-methyltransferase SETD1A | 0.676 | 0.034 | 0.034 | -0.15 | -0.90 | -0.75 |
| Znf638 | Zincfingerprotein 638 | 0.017 | 0.408 | 0.048 | 1.09 | 0.34 | -0.75 |
| Nemf | Ribosome quality control complex subunit NEMF | 0.097 | 0.002 | 0.026 | -0.68 | -1.43 | -0.75 |
| Golim4 | Golgi integral membraneprotein 4 | 0.687 | 0.116 | 0.022 | 0.15 | -0.59 | -0.74 |
| Lta4h | Leukotriene A-4 hydrolase | 0.007 | 0.562 | 0.005 | 0.90 | 0.16 | -0.74 |
| Itga8 | Integrin alpha-8 [Cleaved into: Integrin alpha-8 heavy chain; Integrin alpha-8 light chain] | 0.018 | 0.649 | 0.028 | 0.88 | 0.14 | -0.74 |
| Pdgfrb | Platelet-derived growth factor receptor beta | 0.402 | 0.005 | 0.009 | -0.28 | -1.02 | -0.74 |
| Pld1 | Phospholipase D1 | 0.386 | 0.175 | 0.031 | 0.31 | -0.42 | -0.74 |
| Jcad | Junctionalcadherin 5-associated protein | 0.238 | 0.387 | 0.024 | 0.43 | -0.31 | -0.74 |
| Pde2a | cGMP-dependent 3',5'-cyclic phosphodiesterase | 0.894 | 0.031 | 0.008 | 0.04 | -0.69 | -0.73 |
| Gabpa | GA-bindingproteinalphachain | 0.005 | 0.133 | 0.024 | 1.24 | 0.51 | -0.73 |
| Psmd7 | 26S proteasome non-ATPase regulatory subunit 7 | 0.001 | 0.054 | 0.015 | 1.32 | 0.60 | -0.72 |
| Dctn3 | Dynactinsubunit 3 | 0.680 | 0.127 | 0.024 | 0.15 | -0.57 | -0.72 |
| Map1s | Microtubule-associatedprotein 1S | 0.078 | 0.490 | 0.006 | 0.53 | -0.19 | -0.71 |
| Thoc3 | THO complexsubunit 3 | 0.011 | 0.266 | 0.040 | 1.02 | 0.31 | -0.71 |
| Mgat5 | Alpha-1,6-mannosylglycoprotein 6-beta-N-acetylglucosaminyltransferase A | 0.783 | 0.111 | 0.048 | 0.10 | -0.60 | -0.71 |
| P3h1 | Prolyl 3-hydroxylase 1 | 0.064 | 0.641 | 0.011 | 0.57 | -0.13 | -0.70 |
| Kdm1a | Lysine-specific histone demethylase 1A | 0.025 | 0.225 | 0.047 | 1.29 | 0.59 | -0.70 |
| Tp53bp1 | TP53-binding protein 1 | 0.136 | 0.640 | 0.037 | 0.56 | -0.14 | -0.70 |
| Erc1 | ELKS/Rab6-interacting/CAST family member 1 | 0.410 | 0.357 | 0.042 | 0.33 | -0.36 | -0.69 |
| Gapvd1 | GTPase-activating protein and VPS9 domain-containing protein 1 | 0.064 | 0.787 | 0.012 | 0.61 | -0.08 | -0.69 |
| Sun2 | SUN domain-containingprotein 2 | 0.087 | 0.825 | 0.042 | 0.62 | -0.07 | -0.68 |
| Atp8a1 | Phospholipid-transportingATPase IA | 0.070 | 0.921 | 0.044 | 0.72 | 0.03 | -0.68 |
| Fndc3a | Fibronectin type-III domain-containing protein 3A | 0.209 | 0.558 | 0.026 | 0.48 | -0.20 | -0.68 |
| Ptk7 | Inactivetyrosine-protein kinase 7 | 0.157 | 0.314 | 0.018 | 0.41 | -0.26 | -0.68 |
| Pacs1 | Phosphofurin acidic cluster sorting protein 1 | 0.008 | 0.144 | 0.043 | 1.19 | 0.53 | -0.67 |
| Rbm39 | RNA-bindingprotein 39 | 0.727 | 0.027 | 0.021 | -0.11 | -0.76 | -0.66 |
| Snrpd2 | Small nuclear ribonucleoprotein Sm D2 | 0.015 | 0.831 | 0.014 | 0.71 | 0.05 | -0.66 |
| Cul2 | Cullin-2 | 0.207 | 0.205 | 0.011 | 0.33 | -0.32 | -0.65 |
| Slc38a10 | Putative sodium-coupled neutral amino acid transporter 10 | 0.012 | 0.240 | 0.035 | 1.07 | 0.42 | -0.65 |
| Tlr3 | Toll-like receptor 3 | 0.303 | 0.010 | 0.043 | -0.36 | -1.01 | -0.65 |
| Rps8 | 40S ribosomalprotein S8 | 0.971 | 0.030 | 0.033 | -0.01 | -0.65 | -0.64 |
| Scarb2 | Lysosomemembraneprotein 2 | 0.117 | 0.848 | 0.046 | 0.58 | -0.06 | -0.64 |
| Anapc4 | Anaphase-promotingcomplexsubunit 4 | 0.013 | 0.391 | 0.028 | 0.91 | 0.27 | -0.64 |
| Gnb4 | Guanine nucleotide-binding protein subunit beta-4 | 0.141 | 0.529 | 0.033 | 0.45 | -0.18 | -0.63 |
| Rplp0 | 60S acidic ribosomal protein P0 | 0.050 | 0.710 | 0.016 | 0.54 | -0.09 | -0.63 |
| Bcap29 | B-cell receptor-associated protein 29 | 0.183 | 0.436 | 0.024 | 0.40 | -0.21 | -0.62 |
| Dock7 | Dedicatorofcytokinesisprotein 7 | 0.418 | 0.213 | 0.024 | 0.24 | -0.37 | -0.61 |
| Entpd1 | Ectonucleosidetriphosphatediphosphohydrolase 1 | 0.014 | 0.435 | 0.040 | 0.83 | 0.22 | -0.61 |
| Rpl10 | 60S ribosomalprotein L10 | 0.105 | 0.343 | 0.012 | 0.40 | -0.21 | -0.61 |
| Plscr2 | Phospholipid scramblase 2 | 0.013 | 0.594 | 0.015 | 0.74 | 0.13 | -0.61 |
| Cast | Calpastatin | 0.001 | 0.075 | 0.006 | 0.98 | 0.39 | -0.60 |
| Uaca | Uveal autoantigen with coiled-coil domains and ankyrin repeats | 0.273 | 0.391 | 0.037 | 0.35 | -0.25 | -0.60 |
| Rhob | Rho-related GTP-binding protein RhoB | 0.479 | 0.200 | 0.027 | 0.21 | -0.39 | -0.59 |
| Nfu1 | NFU1 iron-sulfur cluster scaffold homolog, mitochondrial | 0.047 | NA | 0.047 | 0.59 | 0.00 | -0.59 |
| Hsdl1 | Inactive hydroxysteroid dehydrogenase-like protein 1 | 0.002 | 0.157 | 0.009 | 0.94 | 0.35 | -0.59 |
| Sart3 | Squamous cell carcinoma antigen recognized by T-cells 3 | 0.009 | 0.316 | 0.018 | 0.85 | 0.27 | -0.58 |
| Micu1 | Calcium uptakeprotein 1, mitochondrial | 0.503 | 0.007 | 0.029 | -0.18 | -0.76 | -0.58 |
| Cyb5a | Cytochrome b5 | 0.941 | 0.102 | 0.040 | -0.02 | 0.56 | 0.58 |
| Gorasp2 | Golgi reassembly-stackingprotein 2 | 0.390 | 0.241 | 0.025 | -0.25 | 0.35 | 0.60 |
| Macrod1 | ADP-ribose glycohydrolase MACROD1 | 0.053 | 0.002 | 0.045 | 0.71 | 1.32 | 0.61 |
| Ldah | Lipid droplet-associatedhydrolase | 0.849 | 0.073 | 0.048 | 0.07 | 0.68 | 0.62 |
| Cab39 | Calcium-bindingprotein 39 | 0.093 | 0.004 | 0.049 | 0.67 | 1.29 | 0.62 |
| Enah | Protein enabled homolog | 0.311 | 0.008 | 0.021 | 0.32 | 0.95 | 0.63 |
| Mtap | S-methyl-5'-thioadenosine phosphorylase | 0.276 | 0.005 | 0.024 | 0.35 | 1.00 | 0.65 |
| Idh3g | Isocitratedehydrogenase [NAD] subunitgamma 1, mitochondrial | 0.024 | 0.001 | 0.048 | 1.00 | 1.66 | 0.66 |
| Casr | Extracellular calcium-sensingreceptor | 0.008 | <0.001 | 0.034 | 1.01 | 1.67 | 0.66 |
| Stat3 | Signal transducer and activator of transcription 3 | 0.442 | 0.006 | 0.019 | 0.23 | 0.90 | 0.66 |
| Specc1 | Cytospin-B | 0.004 | 0.235 | 0.007 | -0.99 | -0.33 | 0.67 |
| Rab27a | Ras-related protein Rab-27A | 0.006 | <0.001 | 0.023 | 1.11 | 1.78 | 0.67 |
| Nlrx1 | NLR familymember X1 | 0.071 | 0.928 | 0.043 | -0.70 | -0.03 | 0.68 |
| Mtdh | Protein LYRIC | 0.096 | 0.495 | 0.019 | -0.50 | 0.18 | 0.68 |
| Cmc1 | COX assembly mitochondrial protein homolog | 0.010 | <0.001 | 0.038 | 1.17 | 1.86 | 0.69 |
| Mapkapk2 | MAP kinase-activated protein kinase 2 | 0.444 | 0.018 | 0.032 | 0.28 | 0.98 | 0.70 |
| Npepps | Puromycin-sensitive aminopeptidase | 0.035 | 0.554 | 0.050 | -0.92 | -0.22 | 0.71 |
| Gtpbp4 | GTP-bindingprotein 4 | 0.703 | 0.060 | 0.046 | -0.14 | 0.57 | 0.71 |
| Tnpo1 | Transportin-1 | 0.910 | 0.042 | 0.020 | 0.04 | 0.76 | 0.72 |
| Tpt1 | Translationally-controlledtumorprotein | 0.660 | 0.009 | 0.006 | 0.13 | 0.87 | 0.74 |
| Chchd1 | Coiled-coil-helix-coiled-coil-helix domain-containing protein 1 | 0.031 | NA | 0.031 | -0.74 | 0.00 | 0.74 |
| Kng1 | Kininogen-1 [Cleaved into: Kininogen-1 heavy chain; Bradykinin; Kininogen-1 light chain] | <0.001 | <0.001 | 0.009 | 1.74 | 2.48 | 0.74 |
| Krt18 | Keratin, type I cytoskeletal 18 | <0.001 | <0.001 | 0.016 | 1.89 | 2.64 | 0.75 |
| Rwdd1 | RWD domain-containingprotein 1 | 0.792 | 0.015 | 0.025 | 0.09 | 0.85 | 0.76 |
| Rpl18 | 60S ribosomalprotein L18 | <0.001 | 0.013 | 0.005 | -1.60 | -0.83 | 0.77 |
| Eci1 | Enoyl-CoAdeltaisomerase 1, mitochondrial | 0.404 | 0.005 | 0.031 | 0.30 | 1.08 | 0.77 |
| C2cd2l | Phospholipid transferprotein C2CD2L | 0.721 | 0.092 | 0.027 | -0.14 | 0.64 | 0.77 |
| Ppm1h | Protein phosphatase 1H | 0.342 | 0.069 | 0.006 | -0.27 | 0.51 | 0.78 |
| Pon3 | Serum paraoxonase/lactonase 3 | 0.167 | 0.621 | 0.042 | -0.59 | 0.19 | 0.79 |
| Snx7 | Sorting nexin-7 | 0.753 | 0.049 | 0.039 | 0.14 | 0.93 | 0.79 |
| S100a1 | Protein S100-A1 | <0.001 | <0.001 | 0.012 | 1.73 | 2.53 | 0.79 |
| Vbp1 | Prefoldinsubunit 3 | 0.743 | 0.014 | 0.008 | 0.11 | 0.91 | 0.80 |
| Lypla1 | Acyl-protein thioesterase 1 | 0.116 | 0.001 | 0.040 | 0.66 | 1.46 | 0.80 |
| Ssbp1 | Single-stranded DNA-bindingprotein, mitochondrial | 0.597 | 0.219 | 0.046 | -0.24 | 0.57 | 0.80 |
| Trappc5 | Trafficking protein particle complex subunit 5 | 0.098 | 0.684 | 0.027 | -0.68 | 0.13 | 0.81 |
| Pafah1b1 | Platelet-activating factor acetylhydrolase IB subunit beta | 0.008 | 0.175 | 0.040 | -1.45 | -0.63 | 0.81 |
| Mrps35 | 28S ribosomal protein S35, mitochondrial | 0.004 | <0.001 | 0.014 | 1.13 | 1.95 | 0.82 |
| Tmem176b | Transmembrane protein 176B | 0.949 | 0.074 | 0.040 | 0.03 | 0.85 | 0.82 |
| Mmaa | Methylmalonic aciduria type A homolog, mitochondrial | 0.029 | 0.701 | 0.005 | -0.71 | 0.11 | 0.82 |
| Lancl2 | LanC-like protein 2 | 0.019 | 0.704 | 0.013 | -0.95 | -0.12 | 0.83 |
| Adk | Adenosinekinase | 0.054 | <0.001 | 0.003 | 0.57 | 1.41 | 0.84 |
| Tmx3 | Protein disulfide-isomerase TMX3 | 0.002 | 0.085 | 0.021 | -1.43 | -0.59 | 0.84 |
| Rida | 2-iminobutanoate/2-iminopropanoate deaminase | 0.679 | 0.095 | 0.041 | -0.17 | 0.67 | 0.84 |
| Hax1 | HCLS1-associated protein X-1 | 0.892 | 0.036 | 0.018 | 0.05 | 0.90 | 0.85 |
| Rps9 | 40S ribosomalprotein S9 | <0.001 | 0.001 | 0.019 | -2.27 | -1.42 | 0.86 |
| Ide | Insulin-degradingenzyme | 0.024 | 0.415 | 0.041 | -1.25 | -0.39 | 0.86 |
| Ndufs6 | NADH dehydrogenase [ubiquinone] iron-sulfur protein 6, mitochondrial | 0.250 | 0.004 | 0.026 | 0.47 | 1.33 | 0.86 |
| Stau1 | Double-stranded RNA-bindingprotein Staufen homolog 1 | 0.307 | 0.244 | 0.036 | -0.43 | 0.43 | 0.86 |
| Csrp2 | Cysteineandglycine-richprotein 2 | 0.123 | 0.001 | 0.017 | 0.60 | 1.47 | 0.87 |
| Bpnt2 | Golgi-resident adenosine 3',5'-bisphosphate 3'-phosphatase | 0.007 | 0.340 | 0.014 | -1.20 | -0.33 | 0.87 |
| Cldn19 | Claudin-19 | 0.025 | NA | 0.025 | -0.88 | 0.00 | 0.88 |
| Fbln2 | Fibulin-2 | 0.245 | 0.281 | 0.012 | -0.46 | 0.41 | 0.88 |
| Ranbp1 | Ran-specific GTPase-activating protein | 0.284 | 0.228 | 0.014 | -0.41 | 0.47 | 0.88 |
| Slc4a4 | Electrogenicsodiumbicarbonatecotransporter 1 | 0.137 | 0.654 | 0.046 | -0.70 | 0.19 | 0.89 |
| Ptgr2 | Prostaglandin reductase 2 | 0.573 | 0.006 | 0.017 | 0.20 | 1.09 | 0.89 |
| Cpne1 | Copine-1 | 0.004 | 0.079 | 0.048 | -1.87 | -0.97 | 0.89 |
| Hmga1 | High mobility group protein HMG-I/HMG-Y | 0.198 | 0.003 | 0.016 | 0.51 | 1.41 | 0.90 |
| Gsta4 | Glutathione S-transferase A4 | 0.602 | 0.162 | 0.026 | -0.24 | 0.66 | 0.90 |
| Tor1aip1 | Torsin-1A-interacting protein 1 | 0.011 | 0.405 | 0.023 | -1.26 | -0.34 | 0.92 |
| Denr | Density-regulatedprotein | 0.047 | NA | 0.047 | -0.92 | 0.00 | 0.92 |
| Cnbp | CCHC-type zincfingernucleicacidbindingprotein | 0.442 | 0.004 | 0.012 | 0.27 | 1.19 | 0.92 |
| Eif4a1 | Eukaryotic initiation factor 4A-I | 0.011 | 0.618 | 0.017 | -1.10 | -0.17 | 0.93 |
| Rrm1 | Ribonucleoside-diphosphate reductase large subunit | 0.048 | NA | 0.048 | -0.94 | 0.00 | 0.94 |
| Fkbp15 | FK506-binding protein 15 | 0.048 | 0.682 | 0.008 | -0.81 | 0.14 | 0.94 |
| Afg1l | AFG1-like ATPase | 0.281 | 0.003 | 0.015 | 0.47 | 1.42 | 0.96 |
| Lrrc47 | Leucine-rich repeat-containing protein 47 | 0.032 | 0.646 | 0.008 | -0.85 | 0.13 | 0.98 |
| Fkbp8 | Peptidyl-prolyl cis-trans isomerase FKBP8 | 0.024 | 0.901 | 0.005 | -0.94 | 0.04 | 0.98 |
| Patj | InaD-like protein | 0.015 | 0.440 | 0.034 | -1.32 | -0.33 | 0.99 |
| Sf3b4 | Splicingfactor 3B subunit 4 | 0.086 | 0.003 | 0.049 | 1.10 | 2.09 | 1.00 |
| Rnh1 | Ribonucleaseinhibitor | 0.057 | <0.001 | 0.004 | 0.77 | 1.77 | 1.00 |
| Ppp1r7 | Protein phosphatase 1 regulatorysubunit 7 | 0.915 | 0.019 | 0.046 | -0.05 | 0.96 | 1.02 |
| Nfib | Nuclearfactor 1 B-type | 0.327 | 0.112 | 0.037 | -0.46 | 0.56 | 1.02 |
| Arglu1 | Arginine and glutamate-rich protein 1 | 0.011 | 0.589 | 0.007 | -1.24 | -0.22 | 1.03 |
| Akr1a1 | Aldo-keto reductase family 1 member A1 | 0.548 | 0.007 | 0.025 | 0.27 | 1.33 | 1.06 |
| Fech | Ferrochelatase, mitochondrial | 0.866 | 0.019 | 0.014 | -0.07 | 0.99 | 1.06 |
| Rbm22 | Pre-mRNA-splicing factor RBM22 | 0.120 | 0.310 | 0.010 | -0.67 | 0.40 | 1.07 |
| Adhfe1 | Hydroxyacid-oxoacidtranshydrogenase, mitochondrial | 0.012 | <0.001 | 0.008 | 1.12 | 2.20 | 1.07 |
| Mat2b | Methionineadenosyltransferase 2 subunitbeta | 0.109 | 0.526 | 0.045 | -0.86 | 0.23 | 1.09 |
| Msrb2 | Methionine-R-sulfoxide reductase B2, mitochondrial | 0.344 | 0.001 | 0.004 | 0.32 | 1.42 | 1.09 |
| Hagh | Hydroxyacylglutathionehydrolase, mitochondrial | 0.446 | 0.006 | 0.013 | 0.36 | 1.46 | 1.10 |
| Znf800 | Zincfingerprotein 800 | 0.325 | 0.186 | 0.042 | -0.51 | 0.59 | 1.10 |
| Mydgf | Myeloid-derivedgrowthfactor | 0.944 | 0.003 | 0.003 | -0.02 | 1.08 | 1.10 |
| Rps28 | 40S ribosomalprotein S28 | 0.001 | 0.022 | 0.028 | -2.43 | -1.32 | 1.11 |
| Ndufaf2 | NADH dehydrogenase [ubiquinone] 1 alpha subcomplex assembly factor 2 | 0.820 | 0.005 | 0.003 | 0.08 | 1.19 | 1.11 |
| Ldhb | L-lactatedehydrogenase B chain | 0.534 | 0.007 | 0.027 | 0.32 | 1.44 | 1.12 |
| Dstn | Destrin | 0.005 | 0.730 | 0.006 | -1.26 | -0.12 | 1.14 |
| Inmt | Indolethylamine N-methyltransferase | 0.212 | 0.379 | 0.028 | -0.69 | 0.45 | 1.14 |
| Rps7 | 40S ribosomalprotein S7 | 0.034 | 0.487 | 0.048 | -1.60 | -0.46 | 1.14 |
| Phpt1 | 14 kDaphosphohistidinephosphatase | 0.165 | 0.002 | 0.030 | 0.78 | 1.92 | 1.14 |
| Cmpk1 | UMP-CMP kinase | 0.136 | 0.085 | 0.002 | -0.55 | 0.60 | 1.14 |
| Rpa1 | Replication protein A 70 kDa DNA-binding subunit | 0.779 | 0.034 | 0.009 | -0.11 | 1.03 | 1.15 |
| Acsl4 | Long-chain-fatty-acid--CoA ligase 4 | 0.006 | 0.968 | 0.003 | -1.18 | -0.01 | 1.16 |
| Ppm1f | Protein phosphatase 1F | 0.104 | 0.387 | 0.008 | -0.80 | 0.37 | 1.16 |
| Smarcd2 | SWI/SNF-related matrix-associated actin-dependent regulator of chromatin subfamily D member 2 | 0.003 | 0.442 | 0.003 | -1.44 | -0.27 | 1.17 |
| Slc25a3 | Phosphate carrierprotein, mitochondrial | 0.551 | 0.002 | 0.008 | 0.25 | 1.43 | 1.17 |
| Fkbp1a | Peptidyl-prolyl cis-trans isomerase FKBP1A | 0.352 | 0.002 | 0.049 | 0.56 | 1.74 | 1.18 |
| Kmt2a | Histone-lysine N-methyltransferase 2A | 0.011 | 0.404 | 0.019 | -1.57 | -0.36 | 1.21 |
| Pfdn6 | Prefoldinsubunit 6 | 0.857 | 0.002 | 0.001 | 0.06 | 1.28 | 1.22 |
| Calml4 | Calmodulin-like protein 4 | 0.479 | 0.043 | 0.010 | -0.32 | 0.90 | 1.23 |
| Fahd2 | Fumarylacetoacetate hydrolase domain-containing protein 2A | 0.387 | <0.001 | 0.001 | 0.28 | 1.52 | 1.24 |
| Vnn1 | Pantetheinase | 0.992 | 0.052 | 0.039 | 0.01 | 1.25 | 1.24 |
| Thoc7 | THO complexsubunit 7 homolog | 0.194 | 0.160 | 0.030 | -0.69 | 0.56 | 1.25 |
| Dtymk | Thymidylatekinase | 0.805 | 0.010 | 0.019 | -0.12 | 1.13 | 1.25 |
| Echdc2 | Enoyl-CoA hydratase domain-containing protein 2, mitochondrial | 0.868 | 0.007 | 0.001 | -0.06 | 1.19 | 1.25 |
| Pitpnc1 | Cytoplasmicphosphatidylinositoltransferprotein 1 | 0.774 | 0.028 | 0.041 | -0.16 | 1.11 | 1.27 |
| Ssrp1 | FACT complexsubunit SSRP1 | <0.001 | 0.007 | 0.006 | -2.23 | -0.95 | 1.28 |
| Pex14 | Peroxisomalmembraneprotein PEX14 | 0.033 | 0.901 | 0.023 | -1.25 | 0.04 | 1.29 |
| Ak1 | Adenylatekinaseisoenzyme 1 | 0.039 | 0.898 | 0.013 | -1.24 | 0.06 | 1.30 |
| Manba | Beta-mannosidase | 0.199 | 0.001 | 0.004 | 0.61 | 1.91 | 1.30 |
| Ddx39b | Spliceosome RNA helicase Ddx39b | <0.001 | 0.004 | 0.016 | -3.37 | -2.06 | 1.31 |
| Qdpr | Dihydropteridinereductase | 0.696 | 0.025 | 0.004 | 0.24 | 1.57 | 1.33 |
| Gnai3 | Guanine nucleotide-binding protein G | 0.001 | 0.061 | 0.020 | -2.63 | -1.27 | 1.35 |
| Rhot2 | MitochondrialRhoGTPase 2 | 0.087 | 0.244 | 0.006 | -0.89 | 0.48 | 1.37 |
| St14 | Suppressor of tumorigenicity 14 protein homolog | 0.032 | NA | 0.032 | -1.38 | 0.00 | 1.38 |
| Npl | N-acetylneuraminatelyase | 0.091 | 0.599 | 0.027 | -1.15 | 0.26 | 1.41 |
| Palm | Paralemmin-1 | 0.269 | 0.238 | 0.025 | -0.74 | 0.67 | 1.41 |
| Acsm5 | Acyl-coenzyme A synthetase ACSM5, mitochondrial | 0.372 | 0.211 | 0.039 | -0.60 | 0.81 | 1.41 |
| Clmn | Calmin | 0.389 | 0.035 | 0.048 | 0.88 | 2.30 | 1.41 |
| Maged1 | Melanoma-associatedantigen D1 | 0.572 | 0.022 | 0.023 | -0.29 | 1.12 | 1.41 |
| Mat2a | S-adenosylmethionine synthase isoform type-2 | 0.031 | 0.669 | 0.033 | -1.66 | -0.24 | 1.42 |
| Mtnd1 | NADH-ubiquinoneoxidoreductasechain 1 | 0.033 | 0.359 | 0.008 | -1.13 | 0.30 | 1.43 |
| Ndufs4 | NADH dehydrogenase [ubiquinone] iron-sulfur protein 4, mitochondrial | 0.084 | 0.041 | 0.001 | -0.68 | 0.75 | 1.43 |
| Sdhb | Succinate dehydrogenase [ubiquinone] iron-sulfur subunit, mitochondrial | 0.326 | 0.187 | 0.025 | -0.64 | 0.80 | 1.44 |
| Rasgrp2 | RAS guanyl-releasingprotein 2 | 0.027 | 0.720 | 0.012 | -1.32 | 0.13 | 1.45 |
| Timm9 | Mitochondrial import inner membrane translocase subunit Tim9 | 0.370 | <0.001 | <0.001 | 0.27 | 1.72 | 1.45 |
| Cpox | Oxygen-dependentcoproporphyrinogen-III oxidase, mitochondrial | 0.297 | 0.137 | 0.039 | -0.71 | 0.75 | 1.46 |
| Kcnj16 | Inwardrectifierpotassiumchannel 16 | 0.436 | 0.004 | 0.013 | 0.48 | 1.94 | 1.46 |
| Slc5a3 | Sodium/myo-inositolcotransporter | 0.320 | 0.001 | 0.018 | 0.59 | 2.04 | 1.46 |
| Ddx39a | ATP-dependent RNA helicase DDX39A | 0.001 | 0.006 | 0.030 | -3.18 | -1.71 | 1.47 |
| Nfia | Nuclearfactor 1 A-type | 0.636 | 0.045 | 0.017 | -0.30 | 1.18 | 1.48 |
| Atl3 | Atlastin-3 | 0.006 | 0.512 | 0.013 | -1.83 | -0.35 | 1.48 |
| Coa3 | Cytochrome c oxidase assembly factor 3 homolog, mitochondrial | 0.262 | 0.134 | 0.021 | -0.66 | 0.83 | 1.49 |
| Slc25a30 | Kidneymitochondrialcarrierprotein 1 | 0.620 | <0.001 | 0.004 | 0.21 | 1.70 | 1.49 |
| Os9 | Protein OS-9 | 0.016 | 0.614 | 0.012 | -1.83 | -0.32 | 1.50 |
| Apoa4 | Apolipoprotein A-IV | 0.163 | 0.001 | 0.007 | 0.78 | 2.28 | 1.50 |
| Cox5b | Cytochrome c oxidase subunit 5B, mitochondrial | 0.235 | 0.003 | 0.026 | 0.84 | 2.35 | 1.51 |
| Cgn | Cingulin | 0.013 | NA | 0.013 | -1.51 | 0.00 | 1.51 |
| Pcyox1 | Prenylcysteineoxidase 1 | 0.004 | 0.055 | 0.041 | -2.96 | -1.43 | 1.54 |
| Ddah1 | N | 0.013 | 0.286 | 0.001 | -1.14 | 0.41 | 1.55 |
| Nudcd3 | NudC domain-containingprotein 3 | 0.423 | 0.020 | 0.002 | -0.35 | 1.21 | 1.56 |
| Ldhd | Probable D-lactatedehydrogenase, mitochondrial | 0.829 | 0.020 | 0.030 | 0.15 | 1.72 | 1.56 |
| Mrpl18 | 39S ribosomalprotein L18, mitochondrial | 0.225 | 0.057 | 0.002 | -0.59 | 0.98 | 1.57 |
| Pdcd6 | Programmedcelldeathprotein 6 | <0.001 | 0.001 | 0.001 | -3.38 | -1.80 | 1.58 |
| Uchl3 | Ubiquitin carboxyl-terminal hydrolase isozyme L3 | 0.202 | 0.260 | 0.046 | -0.97 | 0.63 | 1.59 |
| Man1b1 | Endoplasmic reticulum mannosyl-oligosaccharide 1,2-alpha-mannosidase | 0.004 | 0.288 | 0.009 | -2.28 | -0.68 | 1.60 |
| Snrk | SNF-related serine/threonine-protein kinase | 0.035 | 0.758 | 0.026 | -1.80 | -0.19 | 1.60 |
| Uqcrb | Cytochrome b-c1 complex subunit 7 | 0.291 | 0.328 | 0.044 | -0.87 | 0.74 | 1.61 |
| Nectin2 | Nectin-2 | 0.032 | 0.544 | 0.046 | -2.16 | -0.55 | 1.61 |
| Vamp3 | Vesicle-associatedmembraneprotein 3 | 0.001 | 0.183 | 0.004 | -2.32 | -0.71 | 1.61 |
| Prpf31 | U4/U6 small nuclear ribonucleoprotein Prp31 | 0.063 | 0.719 | 0.049 | -1.97 | -0.34 | 1.63 |
|  | UPF0461 protein C5orf24 homolog | 0.447 | 0.028 | 0.003 | -0.41 | 1.23 | 1.64 |
| Tut7 | Terminal uridylyltransferase 7 | 0.048 | 0.737 | 0.027 | -1.48 | 0.16 | 1.64 |
| Ndufa13 | NADH dehydrogenase [ubiquinone] 1 alpha subcomplex subunit 13 | 0.012 | 0.329 | 0.028 | -2.28 | -0.64 | 1.64 |
| Acy3 | N-acyl-aromatic-L-amino acid amidohydrolase | 0.102 | 0.089 | 0.011 | -0.93 | 0.73 | 1.65 |
| Sgpl1 | Sphingosine-1-phosphate lyase 1 | 0.037 | 0.216 | 0.001 | -1.08 | 0.58 | 1.66 |
| Lox | Protein-lysine 6-oxidase | 0.864 | 0.047 | 0.040 | 0.15 | 1.81 | 1.66 |
| Taco1 | Translational activator of cytochrome c oxidase 1 | 0.001 | 0.108 | 0.003 | -2.51 | -0.85 | 1.66 |
| Ebp | 3-beta-hydroxysteroid-Delta | 0.014 | 0.746 | 0.010 | -1.89 | -0.20 | 1.69 |
| Srp9 | Signal recognition particle 9 kDa protein | 0.029 | 0.980 | 0.012 | -1.70 | 0.01 | 1.71 |
| Mylpf | Myosin regulatory light chain 11 | 0.386 | 0.233 | 0.042 | -0.82 | 0.97 | 1.79 |
| Coasy | Bifunctionalcoenzyme A synthase | 0.558 | 0.030 | 0.005 | -0.36 | 1.45 | 1.81 |
| Faf2 | FAS-associatedfactor 2 | 0.014 | 0.373 | 0.032 | -2.42 | -0.60 | 1.82 |
| Pip4p2 | Type 2 phosphatidylinositol 4,5-bisphosphate 4-phosphatase | 0.095 | 0.445 | 0.022 | -1.23 | 0.60 | 1.83 |
| Cisd1 | CDGSH iron-sulfur domain-containing protein 1 | 0.649 | 0.001 | 0.003 | 0.25 | 2.08 | 1.83 |
| Atp5md | ATP synthasemembranesubunit K, mitochondrial | 0.001 | 0.134 | 0.006 | -2.81 | -0.97 | 1.84 |
| ATP5IF1 | ATPaseinhibitor, mitochondrial | 0.094 | 0.110 | 0.001 | -1.00 | 0.87 | 1.86 |
| Set | Protein SET | <0.001 | 0.001 | 0.007 | -4.85 | -2.98 | 1.87 |
| Fbln1 | Fibulin-1 | 0.011 | 0.799 | 0.001 | -1.73 | 0.14 | 1.87 |
| Adamtsl4 | ADAMTS-like protein 4 | 0.002 | NA | 0.002 | -1.93 | 0.00 | 1.93 |
| Fxyd2 | Sodium/potassium-transportingATPasesubunitgamma | 0.059 | 0.540 | 0.012 | -1.54 | 0.43 | 1.97 |
| Rplp2 | 60S acidic ribosomal protein P2 | 0.008 | 0.480 | 0.018 | -2.52 | -0.54 | 1.98 |
| Acacb | Acetyl-CoAcarboxylase 2 | 0.002 | NA | 0.002 | -2.01 | 0.00 | 2.01 |
| Hmgb1 | High mobility group protein B1 | 0.003 | 0.734 | 0.001 | -2.27 | -0.21 | 2.06 |
| Ndufa11 | NADH dehydrogenase [ubiquinone] 1 alpha subcomplex subunit 11 | 0.027 | 0.584 | 0.003 | -1.75 | 0.35 | 2.10 |
| Dcakd | Dephospho-CoA kinase domain-containing protein | 0.004 | 0.049 | 0.034 | -3.63 | -1.51 | 2.12 |
| Galk1 | Galactokinase | 0.016 | 0.359 | 0.005 | -1.76 | 0.39 | 2.15 |
| Mpc2 | Mitochondrialpyruvatecarrier 2 | 0.012 | 0.986 | 0.005 | -2.19 | -0.01 | 2.18 |
| Ppia | Peptidyl-prolyl cis-trans isomerase A | 0.019 | 0.740 | 0.006 | -1.96 | 0.23 | 2.19 |
| Spn | Leukosialin | 0.006 | 0.239 | 0.041 | -3.48 | -1.26 | 2.23 |
| Tmpo | Lamina-associated polypeptide 2, isoforms alpha/zeta | 0.020 | 0.842 | 0.011 | -2.43 | -0.18 | 2.25 |
| Gca | Grancalcin | 0.491 | 0.001 | 0.007 | 0.44 | 2.73 | 2.29 |
| Ilkap | Integrin-linked kinase-associated serine/threonine phosphatase 2C | <0.001 | 0.004 | <0.001 | -3.39 | -1.10 | 2.29 |
| Gfer | FAD-linked sulfhydryl oxidase ALR | 0.015 | 0.577 | 0.003 | -2.03 | 0.28 | 2.31 |
| Mink1 | Misshapen-like kinase 1 | 0.001 | 0.230 | 0.003 | -3.03 | -0.70 | 2.33 |
| Txnl1 | Thioredoxin-like protein 1 | 0.046 | 0.081 | <0.001 | -1.33 | 1.04 | 2.38 |
| Tpp1 | Tripeptidyl-peptidase 1 | 0.047 | 0.019 | 0.002 | -1.30 | 1.16 | 2.46 |
| Krt2 | Keratin, type II cytoskeletal 2 epidermal | <0.001 | 0.086 | 0.002 | -4.12 | -1.36 | 2.76 |
| Pfkl | ATP-dependent 6-phosphofructokinase, liver type | 0.249 | 0.182 | 0.010 | -1.39 | 1.49 | 2.88 |
| Cav2 | Caveolin-2 | 0.029 | 0.629 | 0.034 | -3.53 | -0.58 | 2.95 |
| Eif5a | Eukaryotic translation initiation factor 5A-1 | 0.039 | 0.619 | 0.046 | -3.78 | -0.54 | 3.24 |
| Gabarapl2 | Gamma-aminobutyric acid receptor-associated protein-like 2 | 0.298 | 0.110 | 0.033 | -1.51 | 1.74 | 3.25 |
| H2bc14 | Histone H2B type 1-M | 0.021 | 0.303 | 0.001 | -4.66 | 1.77 | 6.43 |
